# Gibberellin dynamics governing nodulation revealed using GIBBERELLIN PERCEPTION SENSOR 2 in *Medicago truncatula* lateral organs

**DOI:** 10.1093/plcell/koae201

**Published:** 2024-07-16

**Authors:** Colleen Drapek, Annalisa Rizza, Nadiatul A Mohd-Radzman, Katharina Schiessl, Fabio Dos Santos Barbosa, Jiangqi Wen, Giles E D Oldroyd, Alexander M Jones

**Affiliations:** Sainsbury Laboratory, University of Cambridge, Cambridge CB2 1LR, UK; Sainsbury Laboratory, University of Cambridge, Cambridge CB2 1LR, UK; Sainsbury Laboratory, University of Cambridge, Cambridge CB2 1LR, UK; Sainsbury Laboratory, University of Cambridge, Cambridge CB2 1LR, UK; Sainsbury Laboratory, University of Cambridge, Cambridge CB2 1LR, UK; Institute for Agricultural Biosciences, Oklahoma State University, Stillwater, OK 73401, USA; Sainsbury Laboratory, University of Cambridge, Cambridge CB2 1LR, UK; Department of Plant Sciences, The Crop Science Centre, University of Cambridge, Cambridge CB3 0LE, UK; Sainsbury Laboratory, University of Cambridge, Cambridge CB2 1LR, UK

## Abstract

During nutrient scarcity, plants can adapt their developmental strategy to maximize their chance of survival. Such plasticity in development is underpinned by hormonal regulation, which mediates the relationship between environmental cues and developmental outputs. In legumes, endosymbiosis with nitrogen-fixing bacteria (rhizobia) is a key adaptation for supplying the plant with nitrogen in the form of ammonium. Rhizobia are housed in lateral root-derived organs termed nodules that maintain an environment conducive to Nitrogenase in these bacteria. Several phytohormones are important for regulating the formation of nodules, with both positive and negative roles proposed for gibberellin (GA). In this study, we determine the cellular location and function of bioactive GA during nodule organogenesis using a genetically encoded second-generation GA biosensor, GIBBERELLIN PERCEPTION SENSOR 2 in *Medicago truncatula*. We find endogenous bioactive GA accumulates locally at the site of nodule primordia, increasing dramatically in the cortical cell layers, persisting through cell divisions, and maintaining accumulation in the mature nodule meristem. We show, through misexpression of GA-catabolic enzymes that suppress GA accumulation, that GA acts as a positive regulator of nodule growth and development. Furthermore, increasing or decreasing GA through perturbation of biosynthesis gene expression can increase or decrease the size of nodules, respectively. This is unique from lateral root formation, a developmental program that shares common organogenesis regulators. We link GA to a wider gene regulatory program by showing that nodule-identity genes induce and sustain GA accumulation necessary for proper nodule formation.

## Teaser

Gibberellin patterning is a key difference between nodule organogenesis and lateral root organogenesis.

## Introduction

Nutrient acquisition is a fundamental problem facing plants in ecosystems and crops. Fixed nitrogen is particularly scarce in tropical and agricultural soils and its availability is a key determinant of plant health (Oldroyd and Leyser 2020). The nitrogen-fixing clade of plants, which include legumes, have adapted to house nitrogen-fixing bacteria within root lateral organs, termed nodules, as a means of overcoming nitrogen scarcity. Understanding how they do so is important for improving nutrient uptake in commercially important legumes as well as in attempts to improve nitrogen acquisition in cereal crops (Jhu and Oldroyd 2023).

Several phytohormones are important for regulating the proper formation and maintenance of nodules. Cytokinin signaling stimulates nodule initiation. Constitutive expression of the cytokinin receptor results in nodule organogenesis in the absence of rhizobia (termed spontaneous nodules; Tirichine et al. 2007) and depletion of receptor expression interferes with nodule organogenesis (Gonzalez-Rizzo et al. 2006; Murray et al. 2007). Cytokinin biosynthesis is further activated by symbiotic interaction with rhizobia (Reid et al. 2017). Exogenous application of cytokinin results in the formation of spontaneous nodules in several legume species, including the model plants *Lotus japonicus* and *Medicago truncatula* as well as a number of other legumes from different evolutionary subfamilies (Heckmann et al. 2011; Gauthier-Coles et al. 2018).

Cytokinin recruits the major organogenesis regulator and transcription factor NODULE INCEPTION (NIN), which is required for nodule formation (Gonzalez-Rizzo et al. 2006; Marsh et al. 2007; Plet et al. 2011; Yoro et al. 2014). NIN functions in the activation of the key transcriptional gene networks essential for nodule organogenesis and nitrogen fixation (Plet et al. 2011). NIN itself is expressed specifically in endosymbiotic conditions, especially in dividing pericycle cells, the epidermis, and nodule primordia, which is regulated by both a proximal promoter and distal enhancer elements (Liu et al. 2019).

In addition to hormonal cues, there are several transcriptional regulators that function in organ development and identity. The LIGHT-SENSITIVE SHORT HYPOCOTYL (LSH) proteins LSH1 and LSH2 act downstream of NIN and function specifically in nodule organogenesis (Lee et al. 2024). Mutants in *lsh* have deformed, multilobed nodules that lack infection and nitrogen fixation (Lee et al. 2024). LSH proteins regulate transcriptional co-activators NODULE ROOT 1 (NOOT1) and NOOT2. Mutants of NOOT1 and NOOT2 lose nodule identity and shape, and ultimately convert to lateral-root-like structures (Couzigou et al. 2012; Magne et al. 2018; Shen et al. 2020). LATERAL ORGAN BOUNDARIES DOMAIN proteins 11 and 16 (LBD11/LBD16) regulate both nodule organogenesis and lateral root development and mutants have a reduction in lateral roots and nodules (Schiessl et al. 2019; Soyano et al. 2019). Other factors such as NF-YA1 regulate both aspects of infection and organogenesis (Laloum et al. 2014; Laporte et al. 2014; Xiao et al. 2014).

Gibberellin (GA) hormones have been increasingly appreciated as having a nuanced but important role in rhizobia-legume endosymbiosis. DELLA proteins, the antagonists of GA signaling that are degraded in the presence of GA, have been found to be required for rhizobial infection in *M. truncatula* as well as endosymbiotic fungal infection in *M. truncatula* and rice (*Oryza sativa*), suggesting a negative role for GA in symbiosis (Floss et al. 2013; Yu et al. 2014; Fonouni-Farde et al. 2016; Jin et al. 2016). In line with this conclusion, treatments with low doses of a GA biosynthesis inhibitor paclobutrazol (PAC) in *M. truncatula* or GA biosynthesis inhibitor uniconazole in *L. japonicus* increase nodule number, and exogenous GA reduces nodule number in both species (Maekawa et al. 2009; Fonouni-Farde et al. 2016; Jin et al. 2016). Overexpression of nondegradable DELLA can rescue nodulation in cytokinin mutants (Fonouni-Farde et al. 2017). On the other hand, a mutant of the biosynthetic enzyme ent-kaurenoic acid oxidase, an enzyme that produces a GA precursor among other molecules, in pea (*Pisum sativum*) is deficient in nodulation, and exogenous GA treatment can increase nodule number in low doses while inhibiting at high doses (Ferguson et al. 2005; McAdam et al. 2018). GA biosynthesis genes in *L. japonicus* and soybean (*Glycine max*) are expressed at the site of nodule organogenesis, and there is a general trend of upregulation of GA-biosynthetic genes across transcriptomes of nodulation (Benedito et al. 2008; Limpens et al. 2013; Roux et al. 2014; Schiessl et al. 2019; Akamatsu et al. 2021; Cervantes-Pérez et al. 2022; Chu et al. 2022; Ye et al. 2022; Gao et al. 2023). Recently, *M. truncatula* mutants deficient in the final stage of GA biosynthesis (GA 3-oxidase) were found to have fewer nodules than their wild-type counterparts (Gao et al. 2023). Some rhizobia can also produce GA themselves, which may give them a fitness advantage post-nodule senescence (Hershey et al. 2014; Nett et al. 2017, 2022). These studies of GA function in nodulation imply both positive and negative effects, suggesting finely tuned control of endogenous GA levels or their distribution in space or time.

GA, as a low abundance hormone, is traditionally detected using mass spectrometry-based techniques which require substantial tissue amounts for reliable detection. For example, GA_4_, the most bioactive form, was detected in *L. japonicus* nodules (Akamatsu et al. 2021) while in *M. truncatula*, the more prevalent but less bioactive GA_1_ was described as below the detection limit in roots and detectable only in shoots (Ueguchi-Tanaka et al. 2007; Fonouni-Farde et al. 2018). As these methods are destructive, average hormone levels across all cell compartments and do not report GA concentrations, *in planta* methods are desirable complements. For example, the activity of promoters driving GA biosynthesis genes has been examined in both *L. japonicus* and soybean around the sites of nodulation, as well as in *M. truncatula* symbiotic roots (Akamatsu et al. 2021; Chu et al. 2022; Gao et al. 2023). However, cellular GA levels result from an ensemble of biochemical steps, several of which are carried out by enzyme or transporter families; expression of individual enzyme expression patterns do not necessarily correspond to GA distribution levels, as is the case for GA biosynthesis genes in the Arabidopsis (*Arabidopsis thaliana*) root (Rizza et al. 2017, 2021).

Förster Resonance Energy Transfer (FRET)-based biosensors such as GIBBERELLIN PERCEPTION SENSOR 1 (GPS1) that interact directly with the ligand of interest allow quantification of cellular dynamics, thus enabling deeper understanding of the regulation and function of ligand levels in vivo (Rizza et al. 2017, 2021). GPS1 is sensitive to nanomolar levels of GAs, shows low reversibility and is thus slow to report GA depletions, and functions by indicating high GA concentrations in solution with a high fluorescence emission ratio in the cytosol or nuclei in the case of nuclear localized GPS1 (nlsGPS1). In soybean, *Agrobacterium rhizogenes*-mediated transient expression of nlsGPS1, showed possible accumulation in nodule primordia cells, in agreement with Liquid-chromatography-Mass Spectrometry detection of GA accumulation in nodules (Akamatsu et al. 2021; Chu et al. 2022), but was limited due to the inclusion of substantial non-nuclear fluorescence.

Here, we use a second-generation FRET biosensor for GA, nlsGPS2 (Griffiths et al. 2024), genetically encoded in stable *M. truncatula* transgenics and analyzed with improved nuclei fluorescence segmentation (Rowe et al. 2022), to visualize cellular GA dynamics in vivo and address where and when GA is important for nodule development and maintenance. The nlsGPS2 biosensor harbors orthogonalizing mutations that invert 2 electrostatic interactions in the sensory domain to limit interaction with endogenous components while maintaining sensor function (Griffiths et al. 2024). These result in increased reversibility and abrogated GA hypersensitivity phenotypes previously observed for *nlsGPS1* expressed in *Arabidopsis* (Griffiths et al. 2024). In early nodule development, we used nlsGPS2 to detect a striking pattern of GA accumulation in dividing cortical cells of nodule primordia that is maintained in the mature nodule apex. We determine GA is key in the early initiation of cell division leading to the nodule primordia, size, and function. This is in stark contrast to the initiation of lateral roots that show little to no GA accumulation during organogenesis. Symbiotic mutants that lose their organ identity and convert to root-like structures lose their GA accumulation pattern. We conclude that GA is a key differentiator between nodule and lateral root identity.

## Results

### A genetically encoded second-generation GA sensor shows a hallmark pattern of GA accumulation during nodule organogenesis

In order to understand the time, place, and levels of cellular GA in the rhizobia-legume symbiosis, we stably transformed *M. truncatula* with a second-generation GA biosensor, nlsGPS2, under the control of the *L. japonicus* ubiquitin promoter. The use of a biosensor permits visualization of bioactive GA at cell resolution and thus offers spatial resolution where other techniques, such as chromatography/mass spectrometry, quantify but do not spatially resolve hormone species. We display the nlsGPS2 segmented nuclei with false coloration corresponding to the emission ratio for each nuclei (Supplementary Fig. S1, see Methods for emission ratio quantification). The nlsGPS2 sensor was optimized for use in Arabidopsis (Rizza et al. 2017, 2021); thus, we confirmed the functionality of the sensor in a new species by quantifying emission ratio changes in roots treated with exogenous bioactive GA species (GA_4_ and GA_3_) in comparison to mock treatments (Supplementary Fig. S1).

**Figure 1.**
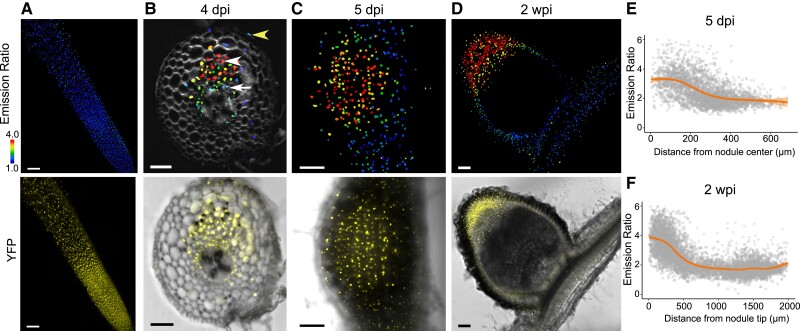
GA accumulates early in nodule development and persists in the nodule apex. **A)** Emission ratio of *LjUBQp:*nlsGPS2 in *M. truncatula* root (top panel) and Yellow Fluorescent Protein (YFP) control (bottom panel), *N* ≥ 20 nodules. **B to D)** Emission ratio of nlsGPS2 and YFP/Brightfield channel overlay in *M. truncatula* nodules inoculated with *Sm2011*. **B)** 4 days post infection (dpi) nodule primordia embedded in 4.5% agarose and sliced in 100 *µ*m sections, *N* = 3 nodules. White arrowhead indicates cortex, white arrow indicates endodermis/stele, yellow arrowhead indicates epidermis. **C)** 5 dpi whole mount nodule primordia, *N* ≥ 20 nodules. **D)** 2 wpi nodule live-embedded 4.5% agarose and sliced in 100 *µ*m sections, *N* ≥ 20 nodules. Images shown are from the central slices of longitudinal nodule sections. **E)** Emission ratio of nuclei (individual dots) from whole mount nodules at 5 dpi as a function of distance from nodule center, *N* = 9 nodules. Curves of best fit are computed in R using a generalized additive model via ggplot. **F)** Quantification of emission ratio of nuclei (individual dots) of 2 weeks post infection (wpi) nodules as a function of distance from nodule tip, *N* = 5. Curves of best fit are computed in R using a generalized additive model via ggplot. Bars = 100 *µ*m in **A, C, D)**; Bar = 50 *µ*m in **B)**. Data shown are from > 3 biological replicates. Additional biological replicates, CFP and FRET channels available in Supplementary Fig. S2.

We then examined nodule development from initiation to maturation over a series of days after inoculation with *Sinorhizobium meliloti* strain 2011 (Sm2011). Prior to infection, GA is low in *M. truncatula* roots (Fig. 1A), but at 4 d post infection (dpi), GA accumulates in dividing cortical cells with low levels in the stele, endodermis and epidermis as observed in radial sections (Fig. 1B, Supplementary Fig. S2). In 5 dpi whole mounted roots, there is a pattern of a high GA gradient decreasing from the center to the periphery of the nodule primordium (Fig. 1C, E). The GA remains low in the root surrounding the developing nodule, an intriguing result given the root-wide expression of GA biosynthesis genes during infection (Gao et al. 2023; Fig. 1C). In order to follow GA accumulation through nodule development and image thick tissue, we live-embedded 2 wpi nodules in agarose and sliced them into thin sections, selecting sections from the largest longitudinal slices through the nodule center. We confirmed this process does not alter GA accumulation by testing it on 5 dpi nodules, which showed a similar GA accumulation pattern in both whole mount and live-embedded samples (Fig. 1C, E; Supplementary Fig. S3). At maturity, GA accumulation remains high toward the nodule apex, but low in the surrounding tissue and stele (Fig. 1D, F), a striking difference from transient nlsGPS1 analysis in soybean (determinate) nodules, where GA was reported to diminish at the nodule apex after several days (Chu et al. 2022).

In addition to confirming the sensor response to GA (Supplementary Fig. S1), it is optimal to test a version of the sensor unable to bind to GA known as nonresponsive (NR) control (Rizza et al. 2017). While all stable lines generated of *LjUBQ:*nlsGPS-NR exhibited some silencing, we were able to image NR controls that had expression persist in nuclei of 5 dpi nodules. These had varied emission ratios but no pattern related to nodule developmental zones (Supplementary Fig. S4).

The same pattern of GA accumulation in earlier stages of nodulation was also detected concomitantly with fluorescently tagged rhizobia forming infection threads into nodule primordia (Supplementary Fig. S5). Note that we do not detect fluorescence of the sensor in the nodule central zone later in infection likely due to the tightly regulated hypoxic environment of infected cells in mature nodules that precludes fluorescent protein maturation (see Discussion).

### The mechanism underlying spatial restriction of GA

**Figure 2.**
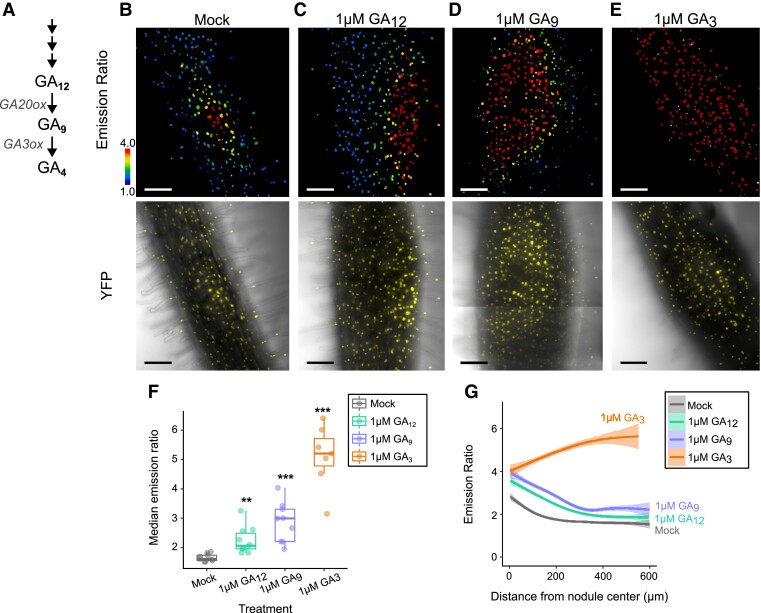
Spatial limitation of GA partially depends on precursor availability. **A)** A simplified schematic of the final metabolic steps in bioactive GA biosynthesis. Enzyme families are italicized. **B to E)** Emission ratio and Yellow Fluorescent Protein (YFP)/Brightfield overlay of nlsGPS2 in developing nodules (5 dpi) treated with **B)** mock (an equal volume of 70% ethanol), **C)** 1 *µ*M GA_12_, **D)** 1 *µ*M GA_9_, and **E)** 1 *µ*M GA_3_, a bioactive GA mimic. Treatment was carried out at time of infection. **F)** Emission ratio by treatment. Each dot represents median emission ratio per sample. Boxplot center line indicates median; box limits, upper and lower quartiles; whiskers, 1.5 × interquartile range. Welch's *t*-test between treatment and mock **P < 0.01, ***P < 0.001. Median values: mock = 1.63, 1 *µ*M GA_12_ = 2.08, 1 *µ*M GA_9_ = 3.01, 1 *µ*M GA_3_ = 5.22. *N* ≥ 7 nodules from three biological replicates. **G)** Emission ratio of nuclei relative by treatment as a function of distance from nodule center. Curves of best fit are computed in R using a generalized additive model via ggplot. *N* ≥ 7 nodules. Bars = 100 *µ*m.

Given the strikingly tight localization of GA accumulation during nodule primordia formation and at maturity, we sought to determine how the spatial accumulation of GA is formed (Fig. 2A). Gibberellin 20-oxidase (GA20ox), which catalyzes the penultimate step in GA biosynthesis, has traditionally been seen as the rate-limiting enzyme in bioactive GA production. However, this does not capture the enzymatic biology relevant in all scenarios (e.g., GA3ox that catalyzes the last biosynthetic step, but not GA20ox, is rate limiting in the *Arabidopsis* root elongation zone; Rizza et al. 2021). We therefore supplied *M. truncatula* roots with the precursors for GA20ox (GA_12_) and GA3ox (GA_9_) to test if either early biosynthetic steps or GA20ox activity were rate limiting for GA accumulation and responsible for GA distribution in nodule development (Fig. 2B to F). Nodules of different treatments were imaged on the same day post infection to synchronize developmental stages. Treatment with GA_12_ at the time of infection quantifiably increased GA within the nodule primordia, but did not alter the generally centralized accumulation of GA (Fig. 2C). Similarly, treatment with GA_9_ quantifiably increased GA levels around the nodule but did not abolish the general pattern of central GA accumulation, indicating that while prior enzymatic steps quantitatively contribute to setting GA levels in nodulation, limitation of GA3ox activity or GA depletion mechanisms exert important control over GA levels and distribution (Fig. 2D, F). Treatment with a bioactive GA, GA_3_, flattens the accumulation across the entire root (Fig. 2E, G). Previously, it was shown that soybean has a nodule-specific GA20ox and that MtNIN can activate the expression of MtGA3ox1 broadly in *M. truncatula* (Chu et al. 2022; Gao et al. 2023). We examined *A. rhizogenes-*transformed roots containing the 3 kb upstream region of MtGA20ox1 (Medtr1g102070) and MtGA3ox1 (Medtr2g102570). In agreement with previously published transcriptome and promoter studies, both genes are broadly expressed in the root (Supplementary Fig. S6). We conclude that the pattern of GA accumulation during nodule development is not defined solely by the expression of the rate-limiting enzymes involved in GA biosynthesis, which are broadly expressed, but rather must also involve posttranscriptional regulation of GA3ox activity or GA depletion (i.e. export and catabolism).

A subset of rhizobia can produce bioactive GA and GA precursors that affect nodule size (Nett et al. 2022), though *S. meliloti* is not annotated to contain the GA biosynthesis gene kaurene synthase (Hershey et al. 2014). We questioned if the initial source of the GA detected with nlsGPS2 (Figs. 1 and 2) could be from rhizobia. To test this, we asked if spontaneous nodules that lack rhizobia contained bioactive GA. We induced spontaneous nodules by introducing a ubiquitously expressed, constitutively active CCaMK into the nlsGPS2 lines via *A. rhizogenes* transformation (Gleason et al. 2006). Spontaneous nodule formation in these lines was low due to both transformation efficiency of *A. rhizogenes* roots in the R108 background and incomplete penetrance of the phenotype (Gleason et al. 2006). Nevertheless, each of 3 spontaneous nodules observed showed GA accumulation (Supplementary Fig. S7), indicating that, at least at nodule onset, GA can be produced by the host.

### GA is required in cortical cells to govern nodule development and size

**Figure 3.**
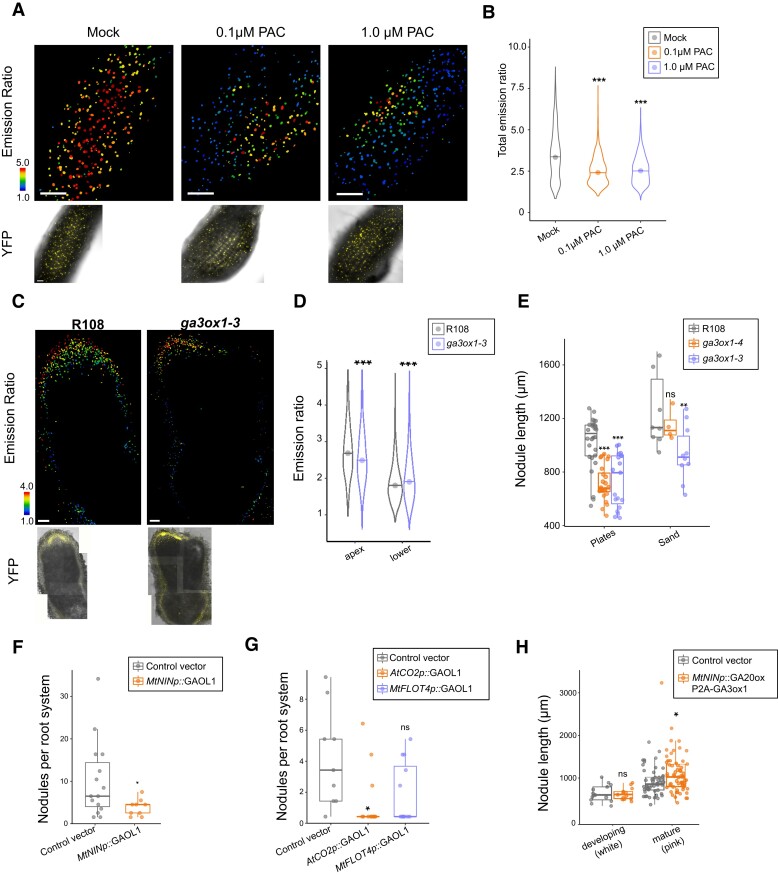
GA functions in early nodule development in cortical cells. **A)** Emission ratio (above) and Brightfield/Yellow Fluorescent Protein (YFP) insets (below) of nlsGPS2 in mock (an equal volume of 70% ethanol) and PAC-treated 5 dpi developing nodules. Treatment was carried out at time of inoculation. **B)** Distribution of emission ratio of nuclei in mock, 0.1 *µ*M PAC and 1.0 *µ*M PAC-treated samples. Mock quantification data is also represented in Fig. 2F-G. Welch's *t*-test between mock and indicated treatment, ***P-value < 0.001. Median values: mock = 3.3445, 0.1 *µ*M PAC = 2.4125, 1.0 *µ*M PAC = 2.5195. *N* ≥ 1,000 nuclei from ≥3 nodules. Representative replicate from three biological replicates is shown. Point represents the mean. **C)** Emission ratio (above) and brightfield/YFP insets (below) of nlsGPS2 in wild-type and *ga3ox1-3* nodules 4 wpi in sand mix. **D)** Distribution of emission ratio of nuclei in the nodule apex and in the lower nodule/root in R108 compared to *ga3ox1-3* in 4 wpi in sand mix. *N* ≥ 1,000 nuclei from ≥5 nodules, Welch's test ***P-value < 0.001. Median values apex: R108 = 2.736, *ga3ox1-3* = 2.56. Median values lower: R108 = 1.851, *ga3ox1-3* = 1.956. Representative replicate from three biological replicates is shown. Point represents the mean. **E)** Nodule length from base to tip at 2 wpi in R108, *ga3ox1*-3 and *ga3ox1-4* on plates or in sand. Boxplot center line indicates median; box limits, upper and lower quartiles; whiskers, 1.5 × interquartile range; all data as points. In the plates assay, Welch's *t*-test ***P-value < 0.001; in the sand assay, Student's *t*-test **P-value < 0.01. Median values plates: R108 = 1087, *ga3ox1-4* = 677, *ga3ox1-3* = 795; median values sand: R108 = 1132, *ga3ox1-4* = 1110, *ga3ox1-3* = 911. **F)** Total nodules per root system in *A. rhizogenes*-transformed roots containing a control construct (*AtUBQp:*AtPIP2A-mScarlet) or the GA-catabolic enzyme *MtGAOL1* under control of a proximal *NIN* promoter containing distal elements. Boxplot center line indicates median; box limits, upper and lower quartiles; whiskers, 1.5 × interquartile range; all data as points. Wilcoxon rank-sum test *P-value < 0.05; *N* ≥ 10 root systems. Median values: control = 5, *MtNINp:*MtGAOL1 = 3. **G)** Total nodules per root system in *A. rhizogenes*-transformed roots containing a control construct (*AtUBQp:* AtPIP2A-mScarlet), the GA-catabolic enzyme *MtGAOL1* under control of the cortex-specific *A. thaliana* promoter *AtCO2* or epidermis-specific promoter *MtFLOT4*. Boxplot center line indicates median; box limits, upper and lower quartiles; whiskers, 1.5 × interquartile range; all data as points. Wilcoxon rank-sum test **P-value < 0.01, *N* ≥ 10 root systems. Median values: control = 3, *AtCO2p*:MtGAOL1 = 0, *MtFLOT4p*:MtGAOL1 = 0. **H)** Nodule length (*µ*m) from base to tip of *A. rhizogenes-*transformed roots containing a control construct (*AtUBQp:* AtPIP2A-mScarlet) or increased local GA biosynthesis (*MtNINp:*GA20ox1-P2A-GA3ox1) in developing (white) nodules and nitrogen-fixing (pink) nodules. Boxplot center line indicates median; box limits, upper and lower quartiles; whiskers, 1.5 × interquartile range; all data as points. Student's *t*-test for developing nodules, not significant. Welch's *t*-test *P-value < 0.05. Median values immature (white): control = 599.687, *MtNINp:*GA20ox1-P2A-GA3ox1 = 609.544; Median values mature (pink): control = 849.228, *MtNINp:*GA20ox1-P2A-GA3ox1 = 1023.115. *N* ≥ 50. Additional *A. rhizogenes* replicates displayed in Supplementary Fig. S10. Bars = 100 *µ*m.

Given the pattern of GA accumulation in nodule primordia, we explored if the accumulation of GA in nodules is functionally important. To do this, we sought to disrupt early accumulation of GA in nodules. We treated plants with 0.1 and 1 *µ*M PAC at the time of infection, which we found, in agreement with previously published reports, resulted in more nodules per plant (Fonouni-Farde et al. 2016; Jin et al. 2016). However, when we examined these nodules for GA accumulation, neither concentration resulted in a lack of GA in developing nodules, though there was a quantifiable decrease in the overall distribution (Fig. 3A, B). Therefore, the increase in nodule number in PAC treatment is not due to a lack of GA in developing nodules and is consistent with a role for DELLA, and therefore GA depletion, elsewhere during initial infection.

We therefore explored a genetic approach to deplete GA using mutants for *MtGA3ox1*, which is highly expressed during the rhizobial symbiosis (Benedito et al. 2008; Limpens et al. 2013; Roux et al. 2014; Schiessl et al. 2019; Cervantes-Pérez et al. 2022; Ye et al. 2022; Gao et al. 2023). We selected 2 insertional alleles for MtGA3ox1, NF19087 (*ga3ox1-3*), and NF13294 (*ga3ox1-4*), which are both located in the first exon (Supplementary Fig. S8). Both are characteristically dwarfed, slow to germinate, and had short internodes, consistent with GA-related phenotypes (Hedden and Thomas 2022). Despite these strong GA-related phenotypes, we observed only subtle effects in nodulation (Supplementary Fig. S9), counter to what has been previously reported for different alleles (Gao et al. 2023). We examined nlsGPS2 in the *ga3ox1-3* background and found GA was still present, indicating this mutant cannot entirely remove GA from the nodule, pointing at likely compensatory genes, for instance, a second copy of GA3ox, *MtGA3ox2* (Medtr1g011580; Fig. 3C, D). While the phenotypes were more subtle than previously reported, we did observe a reduction in nodule size (Fig. 3E), consistent with reports for biosynthetic mutants in pea (Ferguson et al. 2005). Transmission electron microscopy showed symbiosomes appear to have normal morphology in nodules of *ga3ox1* (Supplementary Fig. S9).

To better control GA depletion, we mis-expressed the *GA2-oxidase like (GAOL)* gene *MtGAOL1* (Medtr3g464530), an enzyme in the *Elongated Uppermost Internode-like* family of cytochrome P450 monooxygenases that breaks down bioactive GA. A similar approach using GA-catabolic enzymes has been used in *A. thaliana* for spatially restricting GA (Barker et al. 2021). Misexpression of *MtGAOL1,* under a fully functional *NIN* promoter composed of distal and proximal regions of the promoter (Liu et al. 2019), led to a marked reduction in the total number of nodules and the absence of pink nodules (indicative of the presence of leghemoglobin for functional nitrogen fixation; Fig. 3F, Supplementary Fig. S6). We also expressed *MtGAOL1* under cortical-specific (*A. thaliana CO2*; Rival et al. 2012) and epidermal-specific FLOTILLIN4 (*MtFLOT4*) promoters (Jardinaud et al. 2016; Fonouni-Farde et al. 2017; Liang et al. 2018). Cortical expression of *MtGAOL1* resulted in a severe reduction in developing nodules, whereas epidermal expression of *MtGAOL1* had a trend in nodule reduction but no significant effect (Fig. 3G, Supplementary Fig. S10). Together, these results indicate a role for symbiotic GA accumulation in the behavior of root cortical cells leading to nodule development. To further explore this role, we attempted to locally increase GA levels in the nodule primordia. We expressed both *MtGA20ox1* and *MtGA3ox1* together, under the *NIN* promoter, in *A. rhizogenes-*transformed *M. truncatula* roots. These 2 enzymes are key steps in GA biosynthesis (Hedden 2020), and consistently, we observed quantifiably higher levels of GA in these nodules (Supplementary Fig. S11). While nodule number did not increase in these roots (Supplementary Fig. S11), mature nodule size was significantly larger in mature nodules (Fig. 3H). We conclude that GA is a positive regulator during nodule organogenesis initiation and development.

### GA accumulates downstream of *NIN*

**Figure 4.**
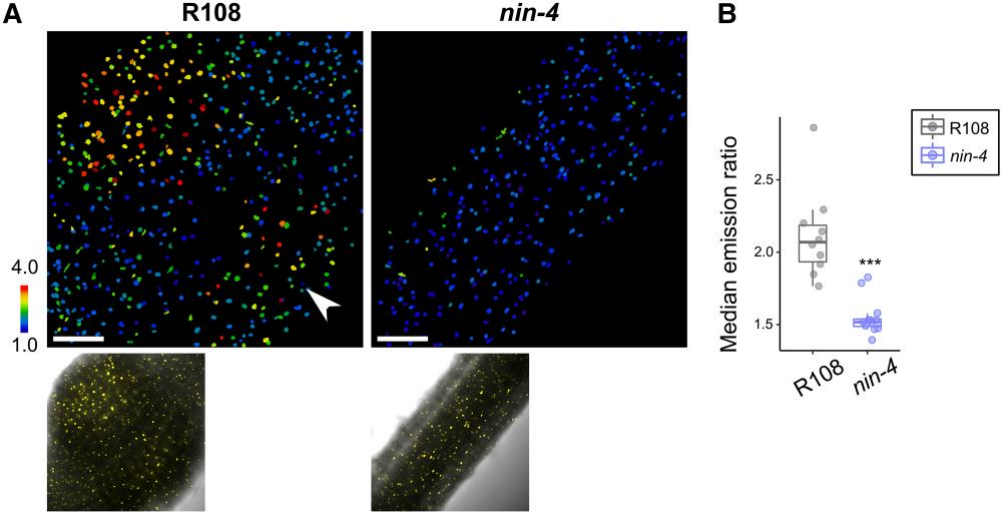
GA accumulates in nodules in a NIN-dependent fashion. **A)** Emission ratio of nlsGPS2 (top panel) and Yellow Fluorescent Protein/brightfield overlay (bottom panel) in R108 (left) and *nin-4* (right) at 5 dpi. Images taken at the point of susceptibility zone at time of infection. Arrowhead indicates second nodule forming. **B)** Median emission ratio from indicated genotype. Boxplot center line indicates median; box limits, upper and lower quartiles; whiskers, 1.5 × interquartile range; all data as points. Each dot represents one sample. Wilcoxon rank-sum test ***P-value < 0.001. Median values: R108 = 2.069, *nin-4* = 1.517. *N* ≥ 10 nodules. Bar = 100 *µ*m. Data shown are from three biological replicates.

Recently, it was shown in *M. truncatula* that the broadly expressed upregulation of *MtGA3ox1* during symbiosis is dependent on NIN (Gao et al. 2023), and therefore, we tested if GA accumulates in the *nin-4* mutant. In line with the conclusions from Gao et al. (2023); we find GA fails to accumulate at the site of inoculation in *nin-4* mutants (Fig. 4)

### GA accumulation is a signature of nodule maturation and is regulated by organ-identity transcription factors

**Figure 5.**
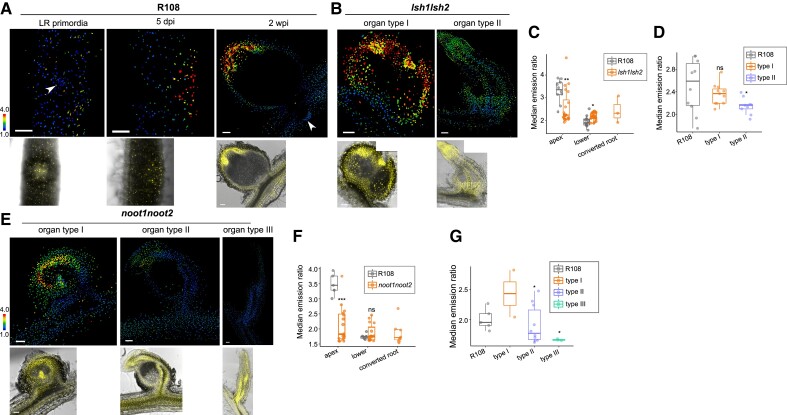
The transcription factors LSH1/2 and NOOT1/2 regulate and maintain GA gradients in mature nodules. **A)** Emission ratio of nlsGPS2 (top panel) and Yellow Fluorescent Protein (YFP)/Brightfield (bottom panel) of an R108 lateral root (LR) primordia (right) from a 5-day-old sample (left), a 5 dpi whole mount nodule (center) and 2 wpi nodule embedded in 4.5% agarose and sliced in 100 *µ*m (right). Arrowheads indicate LR primordia. **B)** Emission ratio of nlsGPS2 (top panel) and YFP/brightfield (bottom panel) of a 2 wpi *lsh1 lsh2* nodule embedded in 4.5% agarose and sliced. Left is type I organ (maintained rounded morphology), and right is a type II organ (a rounded base with a lateral root-like structure). **C)** Median emission ratio of nodule regions from indicated genotypes. Boxplot center line indicates median; box limits, upper and lower quartiles; whiskers, 1.5 × interquartile range; points are median emission from each individual sample. For apex (uppermost region), Wilcoxon rank-sum test, **P-value < 0.01 between R108 and specified genotype, median values: R108 = 3.355; *lsh1 lsh2* = 2.23775. For lower (remainder of nodule below the apex), Student's *t*-test *P-value < 0.05, median values R108 = 1.897, *lsh1 lsh2* = 2.1875. *N* ≥ 9 nodule structures from three biological replicates. **D)** Median emission ratio of entire nodule region from indicated organ type. Boxplot center line indicates median; box limits, upper and lower quartiles; whiskers, 1.5 × interquartile range; points are median emission from each individual sample. Welch's *t*-test *P-value < 0.05, median values: R108 = 2.589, type I = 2.367, type II = 2.16. *N* ≥ 9 nodule structures from three biological replicates. **E)** Emission ratio of nlsGPS2 (top panel) and YFP/brightfield (bottom/right panel) of a 2 wpi *noot1 noot2* nodule embedded in 4.5% agarose and sliced 100 *µ*m sections. Left is type I organ (maintained rounded morphology), center is type II (a rounded base with a lateral root-like structure), and right is type III organ (lateral root-like with a centralized vasculature). **F)** Median emission ratio of nodule regions from indicated genotypes. Boxplot center line indicates median; box limits, upper and lower quartiles; whiskers, 1.5 × interquartile range; points are median emission from each individual sample. For apex (uppermost nodule region), Wilcoxon rank-sum test ***P-value < 0.001, median values: R108 = 3.45, *noot1 noot2* = 1.836. For lower (remainder of nodule below the apex), median values R108 = 1.755, *noot1 noot2* = 1.759. *N* ≥ 5 nodule structures. Welch's *t*-test, ***P-value < 0.001, *N* ≥ 5 nodule structures. **G)** Median emission ratio of entire nodule region from indicated organ type. Boxplot center line indicates median; box limits, upper and lower quartiles; whiskers, 1.5 × interquartile range; points are median emission from each individual sample. For type II, Wilcoxon rank-sum test *P-value < 0.05; median values: R108 = 1.948, *noot1 noot2* = 1.766, *N* ≥ 5 nodule structures. For type III, Welch's *t*-test *P-value < 0.05, median values: R108 = 1.948, *noot1 noot2* = 1.653. *N* ≥ 3 nodule structures. In **A,B,D)** Bar = 100 *µ*m. Data shown are from three biological replicates. Additional examples and additional region label information in Supplementary Figs. S12 and S13.

We noticed lateral roots failed to accumulate GA in both *M. truncatula* and *A. thaliana* (Fig. 5A, Supplementary Fig. S12). This is an intriguing difference as there is overlap in the developmental organogenesis programs of lateral roots and nodules (Schiessl et al. 2019; Soyano et al. 2019). This suggests that GA accumulation acts as an important differentiator between nodule and lateral root development, and as such may be controlled by nodule-specific identity regulators, *NOOT* and *LSH* (Couzigou et al. 2012; Magne et al. 2018; Shen et al. 2020; Lee et al. 2024) We introduced the nlsGPS2 sensor into *noot* and *lsh* and found disturbances in the GA gradients (Fig. 5B to F). Consistent with reported phenotypes, there is a range of nodule structures that occur in these mutant backgrounds (Fig. 5, Supplementary Figs. S13 and S14). We categorized structure by (i) nodule region (nodule apex, lower nodule, or converted root) and (ii) degree of organ conversion (Fig. 5, Supplementary Figs. S13 and S14). For the degree of organ conversion, we considered mutant organs as type I if they maintained rounded morphology, type II if they had both a rounded base and lateral-root-like structure, and type III if they were fully converted with a centralized vasculature similar to lateral roots (only found in *noot1 noot2*). In *lsh1 lsh2* mutants, GA is significantly lower in the nodule apex compared to wild-type plants, and significantly higher in surrounding tissue, implying a general inability to localize GA accumulation (Fig. 5B, C, Supplementary Fig. S14B). Overall GA levels in *lsh1 lsh2* mutants were not significantly different in type I organs compared to wild-type structures but were significantly lower in converted root-like structures (Fig. 5B to D). Similar to *lsh1 lsh2*, there was significantly less GA in nodule apices of *noot1 noot2* mutants, and overall GA was significantly lower in converted organ structures (Fig. 5E to G). Similar to *lsh1 lsh2*, the *noot1 noot2* mutant has a GA distribution that is slightly higher than the wild type in the lower region of nodule structures (Supplementary Fig. S14C). These results underpin the observation that GA accumulation is closely associated with root organ identity, in that as nodules in these mutants convert to lateral roots, we observe an abolition of GA accumulation.

We sought to determine if supplementing GA could help rescue either organ morphology or nitrogen fixation. We treated *noot* mutants with 100 nm GA_4_ at 4 dpi for a further 10 d (for a total of 14 dpi), so as to supply GA only after the initiation of rhizobial infection, a process highly dependent on the action of DELLAs (Fonouni-Farde et al. 2016; Jin et al. 2016). We found no obvious changes in nitrogen fixation or organ conversion, but post-initiation GA treatment was sufficient to rescue nodule size in *lsh1 lsh2* mutants to wild type, a further link between GA levels and nodule size (Supplementary Fig. S15). This implies that, while *LSH* and *NOOT* have a role in regulating GA distribution, they have other functions in organ-identity maintenance that cannot be solely explained by loss of GA accumulation and that GA loss does not alone explain organ conversion.

### GA accumulation functions in maintaining mature nodule morphology and function

**Figure 6.**
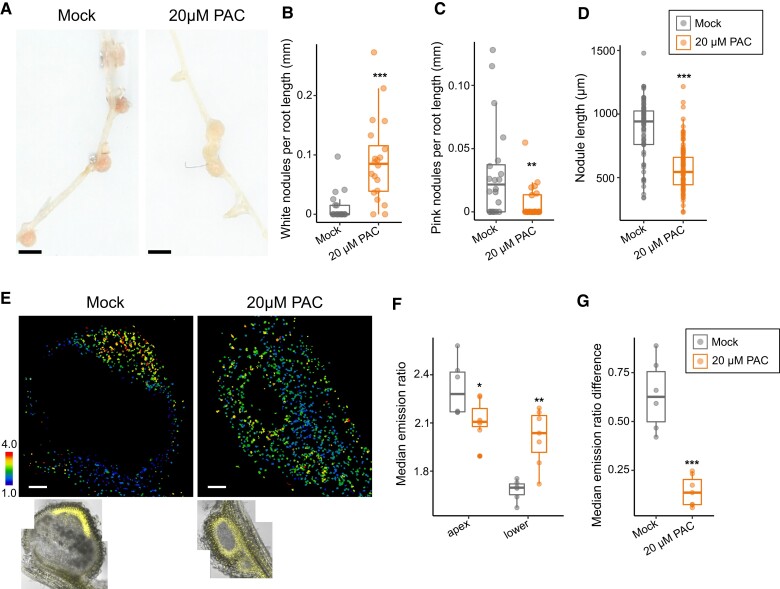
GA patterning is required to maintain nodule growth and nitrogen fixation in mature nodules. **A)** Scanned representative images of 14 dpi nodules from mock (an equal volume of 70% ethanol) treated roots (left) and roots treated with 20 *µ*M PAC for 7 days at 7 dpi. Bar = 1 mm. **B)** Quantification of average white nodules per root length (mm). Boxplot center line indicates median; box limits, upper and lower quartiles; whiskers, 1.5 × interquartile range; all data as points. Welch's *t*-test ***P-value < 0.001, median values mock = 0, PAC = 0.0849. *N* = 20 plants. **C)** Quantification of average pink nodules per root length (mm). Boxplot center line indicates median; box limits, upper and lower quartiles; whiskers, 1.5 × interquartile range; all data as points. Wilcoxon rank-sum test **P-value < 0.01, median values: mock = 0.0215; 20 *µ*M PAC = 0. *N* = 20 plants. **D)** Quantification of nodule length (*µ*m) from mock-treated and 20 *µ*M PAC-treated roots. Boxplot center line indicates median; box limits, upper and lower quartiles; whiskers, 1.5 × interquartile range; all data as points. Welch's *t*-test ***P-value < 0.001, median values: mock = 941.5, 20 *µ*M PAC = 544. *N* ≥ 62 nodules. **E)** Emission ratio images (top panel) and Yellow Fluorescent Protein/Brightfield controls (bottom panel) of a mock-treated nodule and a nodule from roots treated for 7 days with 20 *µ*M PAC. **F)** Emission ratio from nodule apex region or lower nodule/root. Boxplot center line indicates median; box limits, upper and lower quartiles; whiskers, 1.5 × interquartile range; points are median emission from each individual sample. Student's *t*-test for apex region, Welch's *t*-test for lower region, *P-value < 0.05, **P-value < 0.01. Median apex values: mock = 2.294, 20 *µ*M PAC = 2.12. Median lower values: mock = 1.711, 20 *µ*M PAC = 2.051. *N* ≥ 5 nodules. **G)** Absolute value of the median emission ratio difference between the apex and lower region of a nodule. Boxplot center line indicates median; box limits, upper and lower quartiles; whiskers, 1.5 × interquartile range; all data as points. Student's *t*-test ***P-value < 0.001. *N* ≥ 5 nodules. Data shown are from three biological replicates.

We observed GA accumulation at both early initiation, but also in mature nodules, suggesting a possible function for GA throughout nodule development. To test for functions at the later stages of nodule development, we treated roots with high doses of PAC (20 *µ*M) at 7 d after initial infection, and for a further 7 d, so as to not to affect infection or early nodule development. We found treatment with 20 *µ*M PAC significantly decreased GA levels in the mature nodule apex and increased GA levels in the region outside of the nodule (Fig. 6E to G). These roots had a significant decrease in mature nodules, an increase in immature white nodules, and a decrease in overall nodule size (Fig. 6A to D). Taken together, this demonstrate the GA distribution of high in the apex and lower in surrounding tissue functions in post-nodule initiation stages to promote nodule growth and maturation to nitrogen fixation.

## Discussion

The studies of GA function in nodulation in multiple species of legumes present a varied and sometimes confounding picture. Nonetheless, it was clear that GA accumulates during the association with rhizobia and that the DELLA proteins, which are negatively regulated by GA, are positive activators of rhizobial infection. In this study, we use a high-resolution GA FRET biosensor, alongside targeted genetic manipulation, to demonstrate that GA acts as an essential positive regulator of cortical cell division and differentiation required for nodule initiation as well as later nodule growth and maturation. GA accumulation is closely correlated with nodule identity: GA accumulation is a strong differentiator between nodule and lateral root development, and as nodules lose their specific identity, it correlates with a loss of GA accumulation. As such, GA accumulation has opposing functions in the process of nodulation, a positive regulator of cell divisions leading to nodule meristem formation, while also antagonizing rhizobial infection (Fonouni-Farde et al. 2016; Jin et al. 2016). This contrasting functionality in organogenesis versus infection helps explain the confounding results and partly contradictory conclusions of previous studies.

Central to this study is the stable introduction of a GA biosensor into *M. truncatula* that allows an assessment of cellular GA levels throughout the process of nodulation as well as lateral root development. Our results indicate that GA accumulates early during the initiation of cell division in cortical cells, but remains at relatively low levels in the outer cortex and epidermis where rhizobial infection is initiated (Fig. 1B). Such a spatial separation of GA accumulation would allow DELLA accumulation in outer root layers, facilitating rhizobial infection, while at the same time promoting cell divisions in the root cortex. By technical limitation, we do not see the sensor expressed in the central zone of the mature nodule. We theorize this is due to the tight regulation of oxygen in these cells, which is limited for the oxygen-intolerant nitrogenase enzyme (Rutten and Poole 2019). The GFP-derived fluorophores of nlsGPS2 require oxygen for their maturation (Tsien 1998), which may not be readily available in these cells. We can only speculate as to how GA accumulates in these cells, but considering that GA20ox and GA3ox enzymes are 2-oxoglutarate-dependent dioxygenases that require oxygen for catalysis (Hedden and Thomas 2012) and one or both enzymatic steps are likely rate limiting in nodules; GA levels could be either low or dependent on import from other tissues. The enzymes MtGA3ox1 and MtGA3ox2 are lowly expressed in laser capture dissection expression data of the infection zone (Jardinaud et al. 2016). However, expression of individual hormone biosynthesis genes is not a reliable proxy for hormone accumulation (Rizza et al. 2021; Supplementary Fig. S6), and GA biosynthesis genes are often downregulated via negative feedback in response to GA (Hedden and Thomas 2012).

GA accumulation in dividing, cortical cells of the nodule primordia is functionally important for promoting nodule growth and development. Also observed in the mature nodule apex, this unusual association of GA with dividing cells suggests atypical meristem gene regulation in indeterminate nodules. Typically, GA has been linked to cell elongation and cell growth rather than division, for example, in the elongation of hypocotyl cells and shoot internodes (Cowling and Harberd 1999; Zhu et al. 2006). However, in vivo analysis of GA biosynthesis in *Arabidopsis* primary root tips expressing nlsGPS1 revealed distinct enzyme regulatory regimes for apical cells of the division zone versus elongation zone cells (Rizza et al. 2021). Furthermore, targeted manipulation of GA levels in the same tissues indicated that GA promotes division zone length and cell elongation, likely via spatially distinct accumulations (Barker et al. 2021). Lateral meristem identity has also been found to be controlled by GA in grapevine tendrils and strawberry runners, providing useful examples for comparing to the patterns in the nodule apex (Boss and Thomas 2002; Hytönen et al. 2009; Tenreira et al. 2017). GA may also function later in nodule senescence as GA treatment affects this process in *P. sativum* (Serova et al. 2019).

We show the pattern of GA accumulation in nodules is important for governing size and function (Fig. 6), and patterning is downstream of several key components involved in nodule initiation and identity. While bioactive GA accumulation is limited by precursor availability (Fig. 2E and F), spatial restriction of GA does not solely depend on it. This suggests additional layers of tissue regulation, such as localized biosynthetic enzymatic activity, transport, or catabolism. Consistent with this, the catabolic gene *MtGA2ox10* gene has been found to play a role in nodule development and infection thread formation (Kim et al. 2019), and *MtGA2ox2*, another catabolic enzyme, is a top marker gene for nodule vasculature cells in scRNAseq data (Ye et al. 2022), where GA accumulation is low (Fig. 1D). Localized posttranscriptional control of GA-biosynthetic enzymes is an intriguing hypothesis for future studies. Assessing the role of localized transporters is also interesting albeit challenging to address as they are promiscuous and can transport molecules, such as nitrate, in addition to GA (Chiba et al. 2015; Kanno et al. 2016).

We also show that GA does not accumulate in the developing lateral roots of *M. truncatula* (Fig. 5, Supplementary Fig. S12) and that this absence of GA accumulation is shared with *A. thaliana* lateral roots (Supplementary Fig. S12). GA has been reported as a negative regulator of root growth in *M. truncatula* via constitutive expression of GA-catabolic enzymes and chemical treatments (Fonouni-Farde et al. 2019; Kim et al. 2019) and lateral root development is reduced in *della* mutants, which mimic GA (Fonouni-Farde et al. 2019). While a lack of GA accumulation in lateral root primordia is consistent with the hypothesis that GA negatively regulates root development, pleiotropic effects of broad-based perturbations of hormone or hormone signaling suggest root growth effects could be indirect and nonlocal. In the future, it will be interesting to explore these questions with finer-scale perturbations to determine in which cell types and conditions ectopic GA would be impactful for lateral root development.

We show that *NIN* is required for GA accumulation at nodule organogenesis (Fig. 4), a finding consistent with NIN regulation of GA biosynthesis (Gao et al. 2023). The loss of GA patterning in *noot* and *lsh* mutants in nodule maturation is striking and sheds light on their roles as identity regulators, a key component which may be directing and maintaining hormone signaling in mature nodules as a mechanism contributing to their identity roles (Fig. 5, Supplementary Figs. S13 and S14). Consistent with this, recent ChIP data for targets of LSH1 include a set of GA biosynthesis genes, including *MtGA20ox1* (Medtr1g102070), one of the highest expressed GA20ox genes during nodulation, which potentially provides a direct link between LSH signaling and GA regulation (Schiessl et al. 2019; Lee et al. 2024). The rescue in nodule size by GA treatment in *lsh1 lsh2* mutants further supports that the perturbed GA pathway directly affects nodule size maintenance.

We demonstrate that targeted manipulation of GA levels can be leveraged to regulate nodule size, a result that is consistent with a general role for GA in growth and development (Fig. 3E and F), and a discovery that may be of interest in efforts to improve the efficiency of nitrogen fixation. We speculate that there exists spatially resolved accumulations and depletions of GA that explain the concurrent roles of GA and DELLA in the rhizobium symbiosis. Taken together, our results demonstrate the importance of local GA accumulation for nodule primordia development through maturation and highlight how the ability to alter plant hormones in specific tissues potentiates aspects of plant development in diverse plant species.

## Materials and methods

### Plant materials

The nlsGPS2 and nlsGPS-NR were genetically integrated into the R108 background of *M. truncatula* by the crop transformation team at the John Innes Centre. Of the 4 fluorescent lines generated for nlsGPS2, 2 of them had stable, fluorescent expression. Of the 12 fluorescent lines generated for nlsGPS-NR, 2 had expression at the seedling stage and exhibited chimeric silencing after day 3. The NF19087 and NF13294 insertional mutants were ordered from the Oklahoma State University Medicago stock center. Genotyping primers and information can be found in Supplementary Table S1.

### Cloning

The nlsGPS2 (Griffiths et al. 2024) harbors 4 amino acid changes in the sensory domain that result in the inversion of 2 electrostatic interactions to limit interaction with endogenous components while maintaining sensor function. The following mutations were incorporated in constructs designed for legume expression, *AtGID1C* (K13E and K28E) and *AtGAI* (E51K and E54R). The full-length coding sequence (CDS) for nlsGPS2 with domesticated BsaI and BpiI sites was synthesized (GeneWiz) into a Modular Cloning (MoClo) compatible level 0 vector with SC overhangs (AddGene Kit# 1000000047; Engler et al. 2014). This fragment was used with a compatible *L. japonicus UBIQUITIN* promoter (1.118 kb upstream) in a level 0 with PU overhangs and a t35S terminator with ST overhangs. Fragments were assembled into a level 1 vector, followed by assembly with a BASTA resistance cassette and an insulator fragment between the BASTA cassette and sensor following the protocol described in Engler et al. (2014). For cloning of the *LjUBQ:*nlsGPS-NR (Non-Responsive to gibberellin), a full-length CDS with domesticated BsaI and BpiI sites was synthesized (GeneWiz) into a MoClo compatible level 0 vector with SC overhangs (Engler et al. 2014). This fragment was assembled as described for *LjUBQ:*nlsGPS2 above, with the addition of a level 1 construct containing a plasma-membrane-tagged mCherry marker.

For misexpression of *MtGAOL1*, a full-length CDS with domesticated for BsaI and BpiI sites was synthesized (GeneWiz) and amplified with C overhangs. This fragment was combined with a domesticated YPET with S overhangs, a tNOS terminator with ST overhangs and (i) a PU-flanked *MtFLOT4* promoter (2.0 kb upstream), (ii) a PU-flanked AtCO2 promoter (647 bp upstream AT1G62500) or (iii) a synthetic NIN promoter containing the 897 bp containing CE motif flanked by P-sites and the 4.5 kb upstream region flanked by U-sites by GoldenGate reaction into a level 1 vector. For overexpression of GA biosynthesis, the same NIN synthetic promoter described in the former was combined with a domesticated CDS of MtGA20ox1 flanked by S overhangs, a domesticated CDS of P2A flanked by C3 overhangs, and a CDS of *MtGA3ox1* flanked by C4 overhangs, with a 35 s terminator flanked by ST overhangs. These were combined into a level 1 vector by GoldenGate reaction. The promoters of *MtGA20ox1* (Medtr1g102070) and *MtGA3ox1* (Medtr2g102570) were synthesized (Genewiz) flanked by PU overhangs and combined with an NLS-3xVenus cassette flanked by SC overhangs and a 35 s terminator flanked by ST overhangs. These were combined into a level 1 vector by GoldenGate reaction. For induction of spontaneous nodules construct, the *A. thaliana* UBQ10 promoter (1.5 kb upstream) flanked by PU sites was combined with the truncated CDS of MtCCaMKΔ1-311 flanked by SC sites and the *P. sativum* rubisco terminator flanked by ST sites into a level 1 construct. All hairy root level 1 constructs described were assembled into level 2 binary destination vectors that included a selection cassette in position 1 containing a ubiquitously expressed (*A. thaliana* UBQ10) upstream an AtPIP2A CDS (AT3G53420) tagged with mScarlet-i. The control vector included the position 1 cassette described in the former and an insulator cassette in position 2.

#### *A. rhizogenes* hairy root transformation

With the exception of the experiments in Supplementary Fig. S5 using R108 seeds, *M. truncatula* A17 seeds were sterilized with 12% hypochlorite solution for 3 min, rinsed several times with sterile water, and washed for 1 to 4 h at room temperature in sterile water containing 5 *µ*M nystatin. Seeds were incubated for 3 to 6 d on 1% *w*/*v* agar plates containing nitrogen-free Buffered Nodulation Medium (BNM) media (Ehrhardt et al. 1992) containing 0.1 *µ*M GA_4_ and 5 *µ*M nystatin. Seedlings were germinated for 24 h at 24 °C in the dark followed by plating vertically on M-media (0.8 mm KNO_3_,·3.0 mm MgSO_4_,·0.9 mm KCl,·35 *µ*M KH_2_PO_4_,·1.2 mm Ca(NO3)_2_,·30 *µ*M MnCl_2_,·24 *µ*M H_3_BO_3_,·9.2 *µ*M ZnSO_4_,·11.65 nm Na_2_MoO_4_,·0.52 *µ*M CuSO_4_,·40 *µ*M Glycine,·280 *µ*M Myoinositol,·4 *µ*M Nicotinic acid,·0.5 *µ*M Pyridoxine, and·0.3 *µ*M Thiamine·1% Agarose) containing a layer of sterile filter paper. Seeds were sealed with micropore tape and roots were shaded from the light with black plastic. At 2 to 5 days old, seedlings were sliced at the root-hypocotyl junction and swirled in a prepared mix of *A. rhizogenes* (AR1193e). The prepared mix consisted of cultures grown to OD∼1.2, spun down and washed twice with sterile water, and resuspended in sterile water to an OD of 1.0 and a final concentration of 200 *µ*M Acetosyringone. Transformed seedlings were layered with sterile, wet filter paper, and plates were then sealed with parafilm. Seedlings were grown at 20 °C for 1 wk in low light (∼40 *µ*mol/m^2^). After 1 wk, roots were transferred to M-media (see above) containing 250 *µ*g/ml Cefotaxime. For experiments using constructs with tissue-specific constitutive expression of *MtGAOL* or their respective controls, plates were supplemented with 1 *µ*M GA3 to overcome transformation defects from overexpression of GA-catabolic enzymes. After a further 2 wk, the transformation was screened by checking for red fluorescent root systems. Untransformed roots were excised and removed before further experimentation.

#### Inoculation of *M. truncatula* whole and composite plants in sand mix

A mix of 1:1:1 sand: terragreen: vermiculite was prepared in 24T pots. Sand was soaked and mixed with filtered water containing *S. meliloti* Sm2011 containing *nifH:*GUS at an OD = 0.1 Sm2011 was previously grown in minimal media at 28 °C to an OD = 0.8 to 1.0. Sand mixture was spread evenly into pots. Plants were grown in walk-in chambers (22 °C 16 h light; 20 °C 8 h dark; light intensity, 200 *µ*mol/m^2^) for 2 to 4 wk as specified in each experiment, with watering with liquid Fahräeus (Boisson-Dernier et al. 2001) medium the first 2 wk.

#### Inoculation of *M. truncatula* on agar-based plates

*M. truncatula* seeds were sterilized with 12% hypochlorite solution for 3 min, rinsed several times with sterile water, and washed for 1 to 4 h at room temperature in sterile water containing 5 *µ*M Nystatin. Seeds were incubated at 4 °C for 3 to 5 d on BNM media (Ehrhardt et al. 1992) containing 0.1 *µ*M GA_4_ and 5 *µ*M nystatin. Seedlings were germinated for 24 h at 24 °C in the dark followed by plating vertically on BNM media supplemented with 0.1 *µ*M aminoethoxyvinylglycine (AVG) with a piece of sterile filter paper between the media and the seedlings. Plates were sealed with micropore tape and roots were shaded from the dark with black plastic and grown in standard long day conditions (24 °C 16 h light, 22 °C 8 h dark, light intensity 120 *µ*mol/m^2^). After 2 days, plants were spray inoculated with *S. meliloti* Sm2011 containing *nifH*:GUS (OD = 0.025) or Sm2011 ubiquitously expressed mCherry diluted in liquid Fahräeus medium (Boisson-Dernier et al. 2001). The position of the root susceptible zone was marked with a sterile needle at the time of infection. For the chemical treatment of plants, the indicated chemical concentration was added (e.g. GA_3_) or a mock treatment where an equivalent volume of 70% ethanol was used.

### Imaging nlsGPS2 in whole mount roots

For experiments at 4 or 5 days after infection, roots were placed on 10 cm round Petri dishes containing solidified BNM media (without filter paper). The areas around the marked susceptibility zone (which is marked at infection by stabbing a hole in the filter paper with a sterile needle, see above) or around lateral roots for lateral root imaging, were adhered to the plate with 4% to 5% agarose cooled but not yet solidified. After solidification, plates were filled with liquid BNM media. Imaging was carried out with a 25 × water-dipping lens at the spot of interest on an SP8-FLiman (Leica) with HyD detectors. For FRET measurement, the same settings described in Rizza et al. (2017) were applied. Briefly, a 440 nm laser was used for excitement and emission was collected at Cyan Fluorescent Protein (CFP) (425 to 475 nm) and Yellow Fluorescent Protein (YFP) (525 to 575 nm) ranges. For the YFP control channel, a 514 nm laser was used for excitement and YFP (525 to 575 nm) emission was used. The laser intensity for the CFP was set to 5% and 3% for the YFP. The gain was set to a max of 400 for all fluorescent channels. Once set, the gain was kept consistent for each experiment. If needed, multiple views of the same nodule were taken and stitched together (see image processing).

For lateral root experiments with *M. truncatula*, plants were grown on modified Fahräeus medium (Boisson-Dernier et al. 2001) containing 0.5 mm ammonium nitrate. Lateral roots were induced by rotation or removal of the root tip (Norman et al. 2014; Schiessl et al. 2019). Roots were imaged 48 h after lateral root induction using settings described above.

For the lateral root experiments with *A. thaliana*, *p16*:nlsGPS1 seeds in Col-0 background were sterilized, stratified for 48 to 72 h, and plated on ½ MS plant medium without sucrose grown in standard 16 h light conditions (Rizza et al. 2017). Lateral root primordia were imaged at 9 d post sowing using the settings described above.

### Imaging nlsGPS2 in nodule slices/fluorescence microscopy

Live nodules are removed from plants by snipping the root just above and below the nodule. Under a light microscope, 4% to 5% agarose is added to fill a plastic 1 cm mold. Immediately, the nodule was embedded by plunging into the agarose mold with forceps and then oriented as needed for slicing using a fine needle. The mold was allowed to solidify for approximately 2 min, then immediately mounted into a vibratome (Leica), and sliced in 100 *µ*m sections. Between 75% and 100% of the nodule was sliced. Using a light microscope, the largest intact piece representing the most central slice of the nodule was selected for imaging. Samples were immediately imaged after slicing on slides using a 20 × air objective using the excitation and emission settings described above (∼10 min after initial harvesting). For FRET measurement, the same settings described in Rizza et al. (2017) were applied. Briefly, a 440 nm laser was used for excitement, and emission was collected at CFP (425 to 475 nm) and YFP (525 to 575 nm) ranges. For the YFP control channel, a 514 nm laser was used for excitement and YFP (525 to 575 nm) emission was used. The laser intensity for the CFP was set to 5% and 3% for the YFP. The gain was set to a max of 400 for all fluorescent channels. Once set, the gain was kept consistent for each experiment. All images were acquired on the SP8-Fliman with HyD detectors or SP8-iPhox with HyD detectors (inverted). For images acquired on the SP8-iPhox, emission ratios are divided by 3 to be equivalent to those collected on the SP8-Fliman. For embedding nodule primordia, slices were mounted in 0.5% calcofluor white in sterile water to observe cell walls. In a small number of 5 dpi nodules and all 2 wpi nodules/nodule-like structures, multiple views were taken to collect views of the entire organ ensuring a 10% overlap among views for image stitching (see image processing).

### Confocal image analysis and emission ratio quantification

For representative images of nuclei signals, Fiji was used to generate sum stacks and display images (i.e. those not used for quantification) of YFP, CFP, and FRET channels; the channel brightness has been adjusted and the background was removed by assessing the brightness histogram of the background (outside of the root/nodule sample).

For emission ratio quantification, any images of organs containing multiple views were stitched together by Imaris Stitcher (Imaris) and exported as an original resolution TIFF file. For analyzing the nlsGPS2, the FRETENATOR2 plugin was used in FIJI for all emission ratio analyses (Rowe et al. 2022). Briefly, nuclei segmentation was processed using the YFP control channel with the following settings: auto-segmentation “otsu” with small Gaussian sigma = 0.8, large sigma = 4.0; size exclusion minimum ROI size = 10; watershed object splitting on, background subtraction = off. The emission ratio was calculated for each segmented nuclei by evaluation of the signal of the FRET channel divided by the signal from the CFP channel. This is automatically generated as .csv table by FRETENATOR. Saturated nuclei, nonsegmented nuclei, or nuclei too dim in donor CFP channel (sum CFP brightness must be 5% higher than the background) are excluded from analysis by filtering the FRETENATOR Emission Ratio outcome table in R. The maximum projection of the segmented nuclei is false-colored and automatically produced by the FRETENATOR plugin. The range of emission ratio is displayed in each figure (usually 1 to 4). For determining the nodule center, bright field images were observed for *x* and *y* positions blinded from the emission ratio outcome. The distance from this position is calculated in R and used to plot against the emission ratio using ggplot2. Best fit curve is plotted using the generalized additive model function (gam) in ggplot2.

For analyzing the nlsGPS-NR, FRETENATOR was applied with the following settings: auto-segmentation “otsu” with small Gaussian sigma = 0.8, large sigma = 4.0; size exclusion minimum ROI size = 10, maximum ROI size = 1,000; watershed object splitting on, background subtraction = local label based. All FRETENATOR outputs were used to filter data in the following manner: the CFP and YFP channels were slowly scanned through to confirm nuclei properly segmented from autofluorescence; the FRETENATOR-generated Label ID (which labels each individual segmented object) was determined to determine the Label ID of nuclei; only nuclei that were segmented separately from cell walls were selected; the corresponding emission ratio of those objects was identified in the FRETENATOR-generated emission ratio output file; the *x* and *y* position from the nodule center versus the emission ratio was plotted for the identified nuclei.

For normalized emission ratio in plotting distribution, the variance among individual plants was accounted for by subtracting the emission ratio minimum and dividing by the emission ratio maximum minus the emission ratio minimum on a per image basis (Emission ratio − Emission ratio minimum)/(Emission ratio maximum − Emission ratio minimum). This was calculated in R and plotted using ggplot2.

### Transmission electron microscopy

TEM was carried out on 4 wpi nodules inoculated in sand mix with Sm2011. Protocol is described by Lee et al. (2024).

### Image analysis for assessing nodule length

For measuring nodule length from base to tip, images were taken and measured on a VHX-7000 (Keyence) or on a DS-7000 Flatbed scanner (EPSON). For images shown from the flatbed scanner, the image background was removed in Fiji (50-pixel rolling ball radius).

### Plotting and statistics

R software was used for generating all plots and statistics. For statistical tests, data were tested for normality distribution by Shapiro–Wilkes test. For normally distributed data, an *F*-test was used to assess variance. If groups had equal variance, a Student's *t*-test was used to evaluate samples. If the *F*-test had a *P*-value < 0.05, Welch's 2-sample *t*-test was used. For nonnormally distributed data, a Wilcoxon rank-sum test was used, unless there was a sample size of *N* > 20, in which case a Welch's 2-sample test was used. For the normalization emission ratio, a Student's *t*-test was used. All *P*-values and test descriptions can be found in Supplementary Data Sets S1 and S2.

### Accession numbers

The following gene IDs are associated with this manuscript: MtGA20ox1—Medtr1g102070; MtGA3ox1—Medtr2g102570; MtGA3ox2—Medtr1g011580; MtGAOL1—Medtr3g464530.

## Supplementary Material

koae201_Supplementary_Data

## Data Availability

Biological data have been deposited in Apollo Open Access Cambridge Data Repository DOI: 10.17863/CAM.110643.

## Supplementary data

The following materials are available in the online version of this article.

**Supplementary Figure S1.** Response of genetically encoded GA sensor in *M. truncatula*.

**Supplementary Figure S2.** Additional examples of GA accumulation in nodule development. Supports Main Fig. 1.

**Supplementary Figure S3.** Live section of 5 dpi nodules. Supports main Fig. 1.

**Supplementary Figure S4.** Nonresponsive control sensor (NR) shows no pattern of accumulation in 5 dpi nodules. Supports Main Fig. 1.

**Supplementary Figure S5.** Additional examples of GA accumulation in nodule development with fluorescently tagged Sm2011. Supports Main Fig. 1.

**Supplementary Figure S6.** Expression of GA biosynthesis genes in *A. rhizogenes*-transformed *M. truncatula* roots. Supports Main *Fig. 2.*

**Supplementary Figure S7.** GA accumulation in *A. rhizogenes*-transformed spontaneous nodules. Supports Main Fig. 2.

**Supplementary Figure S8.** Schematic of T-DNA insertions in MtGA3ox1 mutants. Supports Main Fig. 3.

**Supplementary Figure S9.** Nodule number and symbiosome phenotypes of *ga3ox1* mutants. Supports Main Fig. 3.

**Supplementary Figure S10.** Supporting information for the function of GA in developing nodule primordia cortical cells. Supports Main Fig. 3.

**Supplementary Figure S11.** Increasing GA in the nodule increases nodule size but not nodule number. Supports Main Fig. 3.

**Supplementary Figure S12.** GA accumulation is low in lateral roots in *M. truncatula* and *A. thaliana.* Supports Main Fig. 5.

**Supplementary Figure S13.** Additional examples of GA accumulation in nodule development in organogenesis mutants. Supports Main Fig. 5.

**Supplementary Figure S14.** Nodule organogenesis mutants have different GA accumulation from wild-type nodules. Supports Main Fig. 5.

**Supplementary Figure S15.** GA treatment after nodule initiation of wild-type and organogenesis mutants. Supports main Fig. 6.

**Supplementary Table S1.** Primer sequences for genotyping.

**Supplementary Data Set 1.** Statistical test details and *P*-values for main figures.

**Supplementary Data Set 2.** Statistical test details and *P*-values for supplementary figures.

## Dive Curated Terms

The following phenotypic, genotypic, and functional terms are of significance to the work described in this paper:

LSH1 Gramene: AT5G28490
LSH1 Araport: AT5G28490

UBQ10 Gramene: AT4G05320
UBQ10 Araport: AT4G05320

LSH2 Gramene: AT3G04510
LSH2 Araport: AT3G04510

CYTOKININ CHEBI: CHEBI:23530

GA3ox1 Gramene: At1g15550
GA3ox1 Araport: At1g15550
