# Supplementary material for: Gibberellin dynamics governing nodulation revealed using GIBBERELLIN PERCEPTION SENSOR 2 in Medicago truncatula lateral organs
Source: Plant Cell. 2024 Jul 16;36(10):4442–56. doi: 10.1093/plcell/koae201 (PMC11449112; doi:10.1093/plcell/koae201)
Supplement: koae201_Supplementary_Data [file koae201_supplementary_data.zip › Drapek_2024_Supplementv2.pdf]

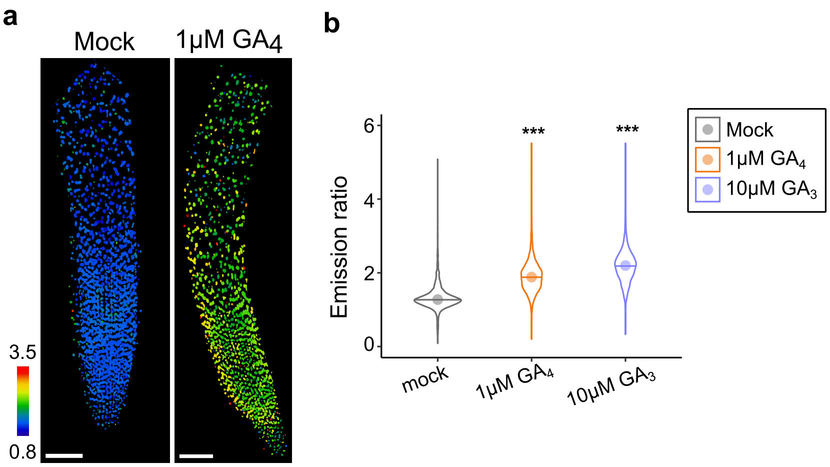

Supplementary Figure S1. Response of genetically-encoded GA sensor in *M. truncatula.* Supports Main Figure 1.

(a) Emission ratio of *M. truncatula* roots containing genetically-encoded nlsGPS2 inoculated for 20 min with mock or 1µM GA_4. Bar = 100 µm. (**b**) Emission ratio distribution of nuclei from roots treated for 20 min with mock (equal volume of 70% ethanol), 1µM GA_4 or 10µM GA_3. N≥3. Welch's t -test ***p-value < 0.001. Median values: mock = 1.274, 1 µM GA_4 = 1.885, 1µM GA_3 = 2.197.

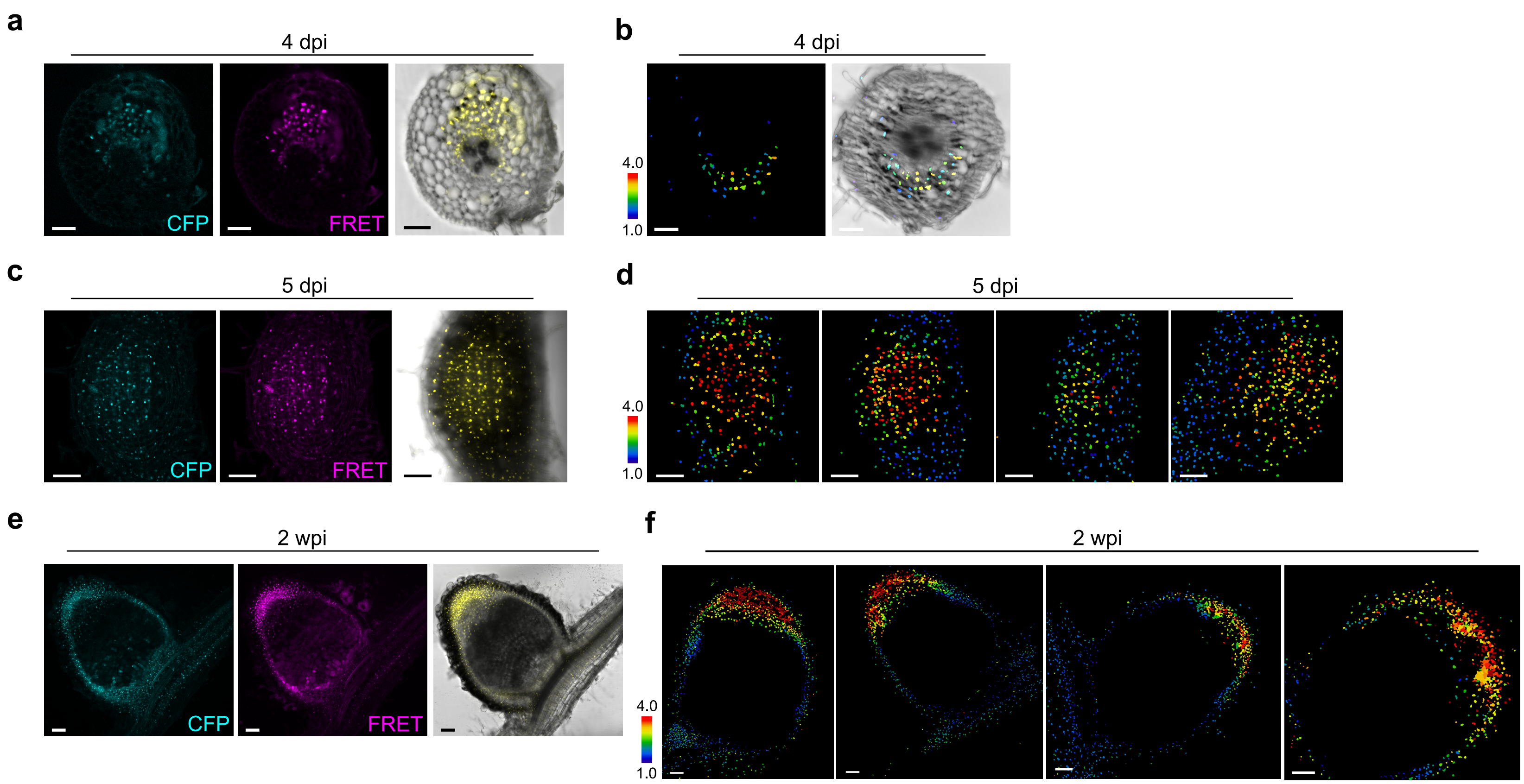

Supplementary Figure S2. **Additional examples of GA accumulation in nodule development. Supports Main Figure 1.** **(a)** Sum projection of cyan fluorescent protein (CFP), Förster resonance energy transfer (FRET) and brightfield/yellow fluorescent protein (YFP) channels from sample in Fig. 1b. **(b)** Emission ratio and YFP/brightfield overlay of additional example of 4 days post infection (dpi) nodule primordia embedded in 4.5% agarose and sliced in 100 µm sections. **(c)** Sum projection of CFP, FRET and brightfield/YFP channels from sample in main Fig. 1c. **(d)** Additional examples of emission ratio of 5 dpi nodule primordia, wholemount. **(e)** Sum projection of CFP, FRET and brightfield/YFP channels from sample in main Fig. 1d. **(f)** Additional examples of emission ratio of 2 weeks post infection (wpi) nodule primordia, embedded in 4.5% agarose and sliced in 100 µm sections. Bar = 100 µm.

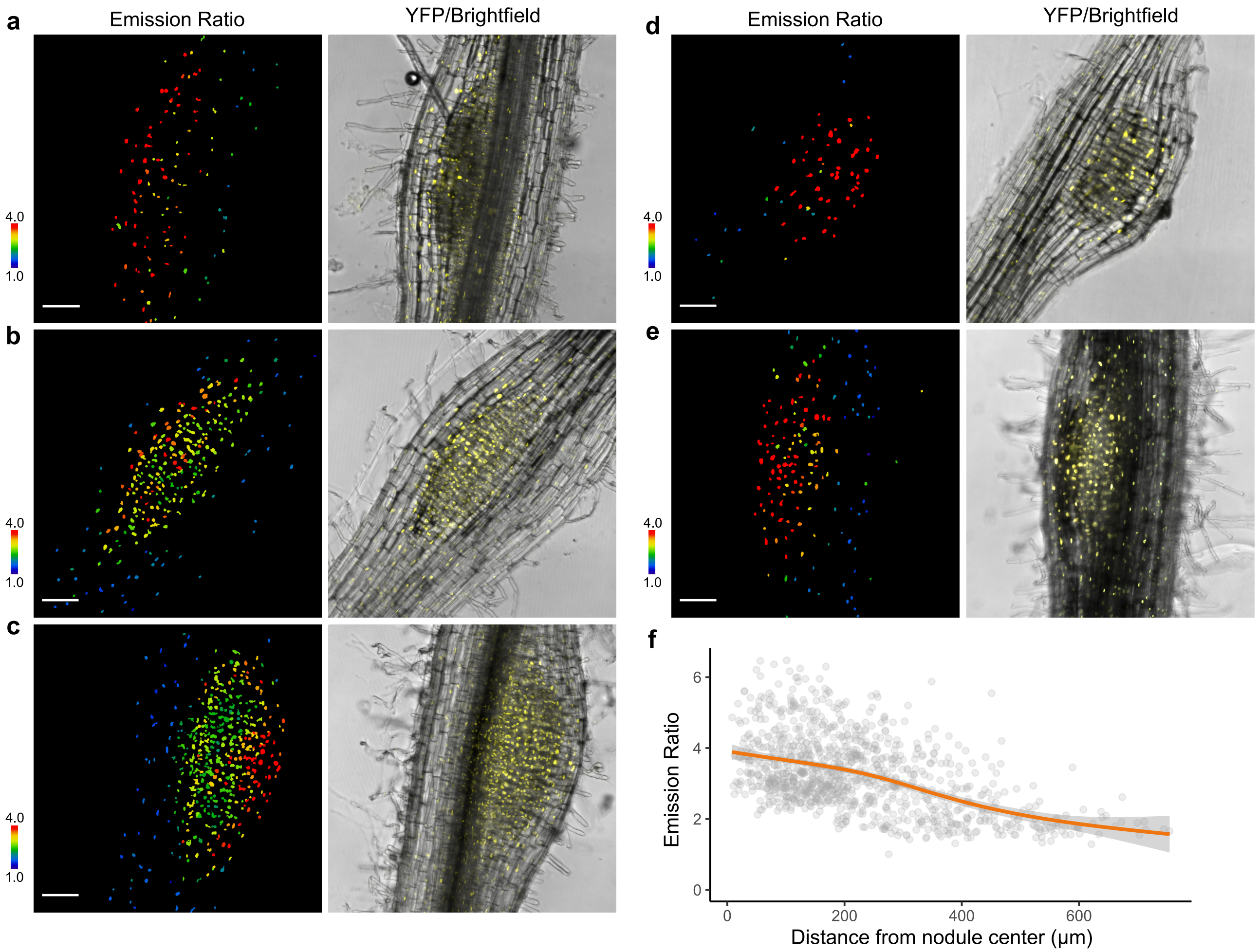

Supplementary Figure S3. Live section of 5 dpi nodules. Supports Main Figure 1.
(a-e) Emission ratio and yellow fluorescdent protein (YFP)/brightfield overlay of examples of *LjUBQ:nlsGPS2* nodules at 5 dpi that were live embedded in 4% agarose and sliced in 100 μm sections (see methods). **(f)** Quantification of emission ratio plotted by distance from nodule center. Bar = 100 μm.

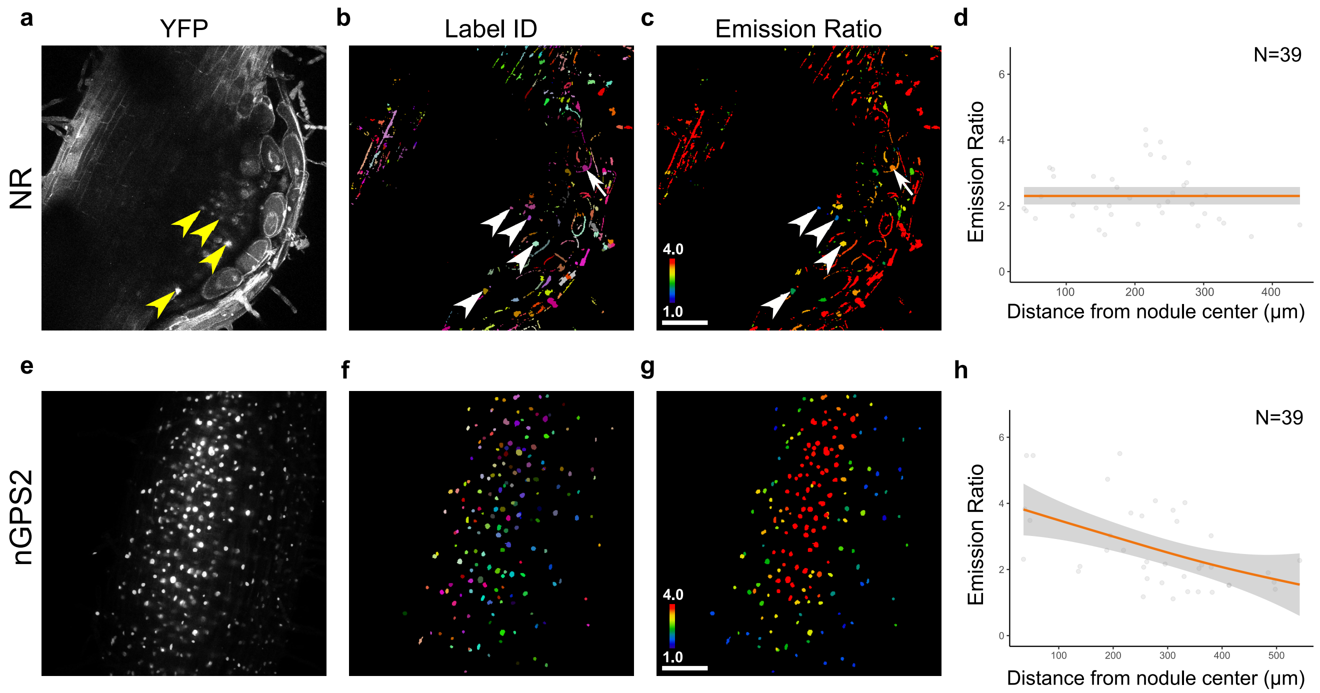

Supplementary Figure S4. Non-responsive control sensor (NR) shows no pattern of accumulation in 5 dpi nodules. Supports Main Figure 1.

(**a**) Sum projection of the YFP channel of a 5 dpi nodule with genetically-encoded expression of the non-responsive control (NR) *LjUBQ:*nlsGPS-NR. Yellow arrowheads indicate examples of nuclei within the nodule. (**b**) A max projection of the Label ID channel of the same nodule in (**a**) generated by FRETENATOR after segmentation. Each differently segmented object is shown in a different color and has an associated emission ratio value. The white arrowheads indicate the same nuclei identified in panel (**a**). The white arrow indicates an example of a nuclei segmented with the surrounding cell wall. (**c**) False-colored max projection of emission ratio of the same nodule in (**a-b**). Arrowheads indicate the same nuclei in (**a-b**) that were properly segmented as individual objects. Arrow indicates nuclei that are excluded due to co-segmentation with surrounding cell wall. (**d**) Quantification of emission ratio compared to distance from nodule center. N = 39 nuclei from three plants.

Panels (**e-h**) present a comparison example to *LjUBQ:*nlsGPS2. (**e**) A sum projection yellow fluorescent protein (YFP) channel, (**f**) the label ID channel and (**g**) max projection of emission ratio as generated by FRETENATOR from a 5 dpi nodule of *LjUBQ:*nlsGPS2. (**h**) A plot of emission ratio versus nodule distance from a random selection of N = 39 nuclei from three plants. Random selection was carried using the sample function in R three times with similar results. Curves in (**d,h**) of best fit are computed in R using a generalized additive model via ggplot. Bar = 100 µm.

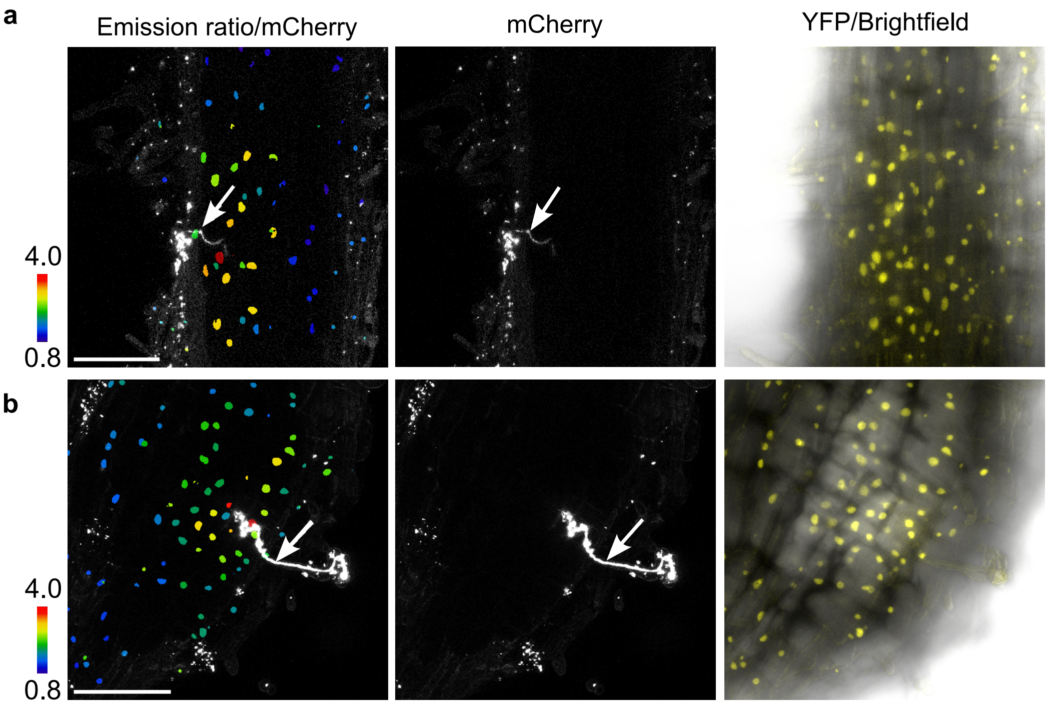

Supplementary Figure S5. Additional examples of GA accumulation in nodule development with fluorescently-tagged Sm2011. Supports Main Figure 1.

(a) Emission ratio and mCherry (Sm2011) merge, mCherry (Sm2011) channel alone, and yellow fluorescent protein (YFP) control/Brightfield overlay. **(b)** Second representative root example. N=5 across three biological replicates. Scale bar = 100 µm, arrows indicate infection threads.

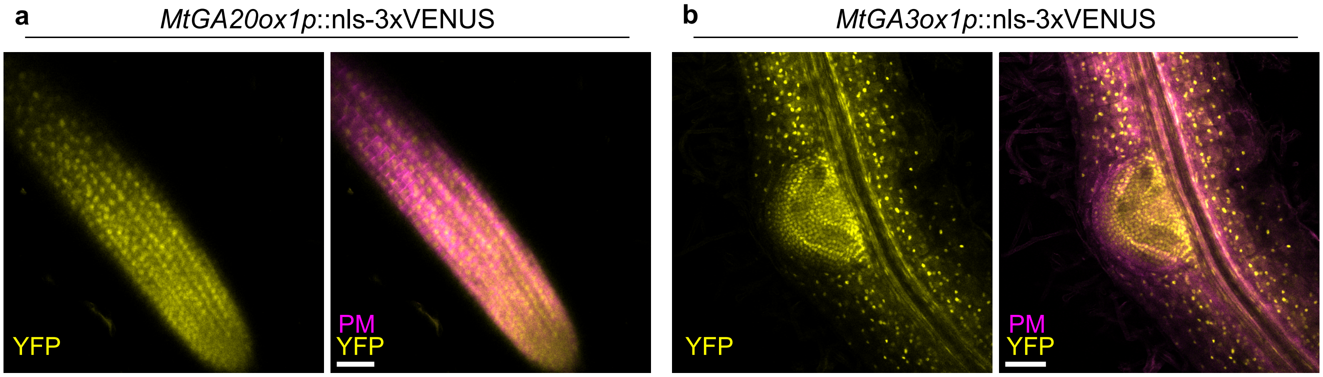

Supplementary Figure S6. Expression of GA biosynthesis genes in *A. rhizogenes*-transformed *M. truncatula* roots. Supports Main Figure 2.

(a) Expression of *MtGA20ox1(3kb)p:nls*-3xVenus in the primary root, N=10. **(b)** Expression of *MtGA3ox1p(3kb)p:*nls-3xVenus in the primary root and lateral root primordia, N=10. PM = ubiquitous plasma-membrane (PM) marker tagged with mScarlet (*AtUBQp:*AtPIP2A-mScarleti) present in vector used for selection. Bar = 50µm.

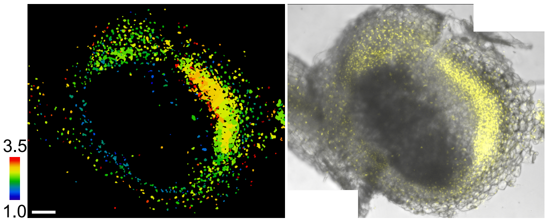

Supplementary Figure S7. GA accumulation in *A. rhizogenes*-transformed spontaneous nodules. Supports Main Figure 2.

Emission ratio image (left) and YFP/Brightfield overlay of *LjUBQp*:nlsGPS2 *M. truncatula* transformed with *AtUBQ10:*MtCCaMKΔ1-311. Spontaneous nodules form after 5 weeks. N=3 nodules. Bar = 100µm.

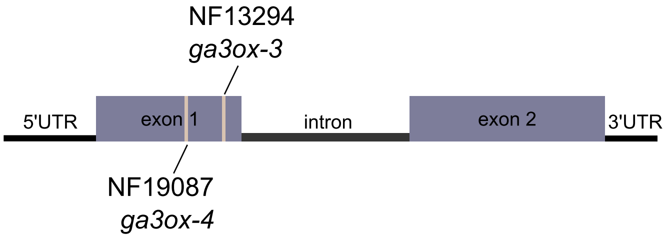

Supplementary Figure S8. Schematic of T-DNA insertions in MtGA3ox1 mutants. Supports Main Figure 3.

Schematic of T-DNA insertions in the first exon of MtGA3ox1 (Medtr2g102570). NF19087 is in the reverse orientation and NF13294 is in the forward orientation. Schematic is to scale.

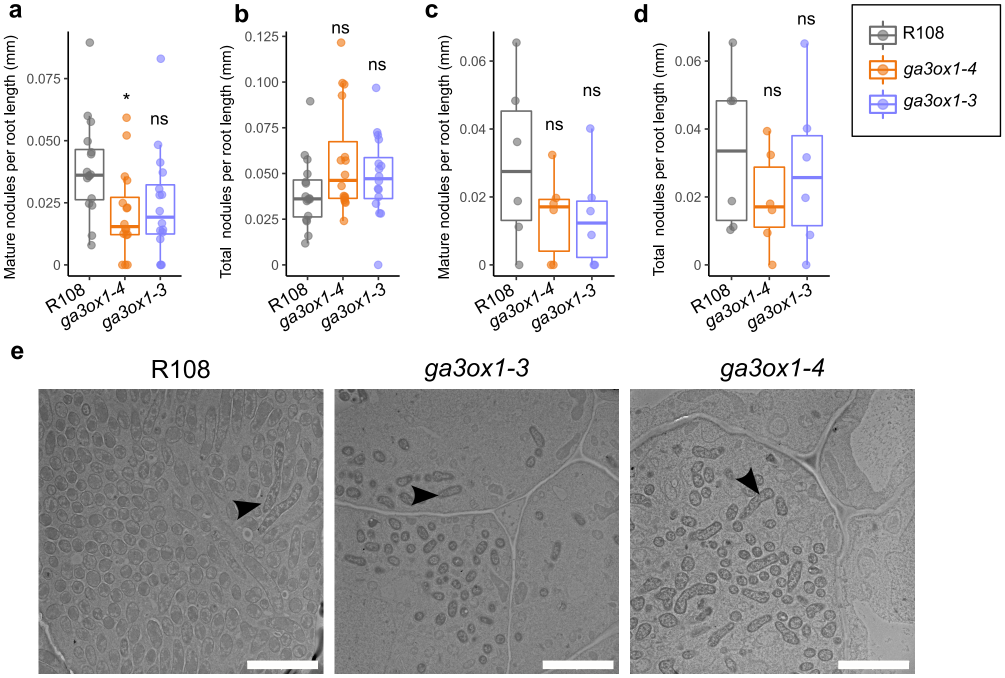

Supplementary Figure S9. Nodule number and symbiosome phenotypes of *ga3ox1* mutants. Supports Main Figure 3.

Quantification of **(a)** mature (pink) nodules and **(b)** total nodules per root length (mm) for R108, *ga3ox1-3* and *ga3ox1-4* inoculated with *S. meliloti* Sm2011 on plates for 14 dpi. Boxplot center line indicates median; box limits, upper and lower quartiles; whiskers, 1.5x interquartile range; all data as points. N=16 plants, Wilcoxon rank sum test for *ga3ox1-4*, Student's t-test for *ga3ox1-3*; p-value * < 0.05. Median values in (a): R108 = 0.036, *ga3ox1-4* = 015, *ga3ox1-3* = 0.019. Median values in (**b**): R108 = 0.036, *ga3ox1-4* = 0.0462, *ga3ox1-3* = 0.0471. Quantification of **(c)** mature (pink) and **(d)** total nodules per root length (mm) for R108, *ga3ox1-3* and *ga3ox1-4* inoculated with Sm2011 in sand mix for 14 dpi. Boxplot center line indicates median; box limits, upper and lower quartiles; whiskers, 1.5x interquartile range; all data as points. N≥6 plants Student's t-test, not significant. Median values in (c): R108 = 0.027, *ga3ox1-4* = 0.017, *ga3ox1-3* = 0.012. Median values in (d): R108 = 0.03, *ga3ox1-4* = 0.017, *ga3ox1-3* = 0.026. (**e**) TEM images 2600x of apical infection zone of R108, *ga3ox1-3* and *ga3ox1-4.* Bar = 5μm. Black arrowheads indicate examples of sectioned symbiosomes.

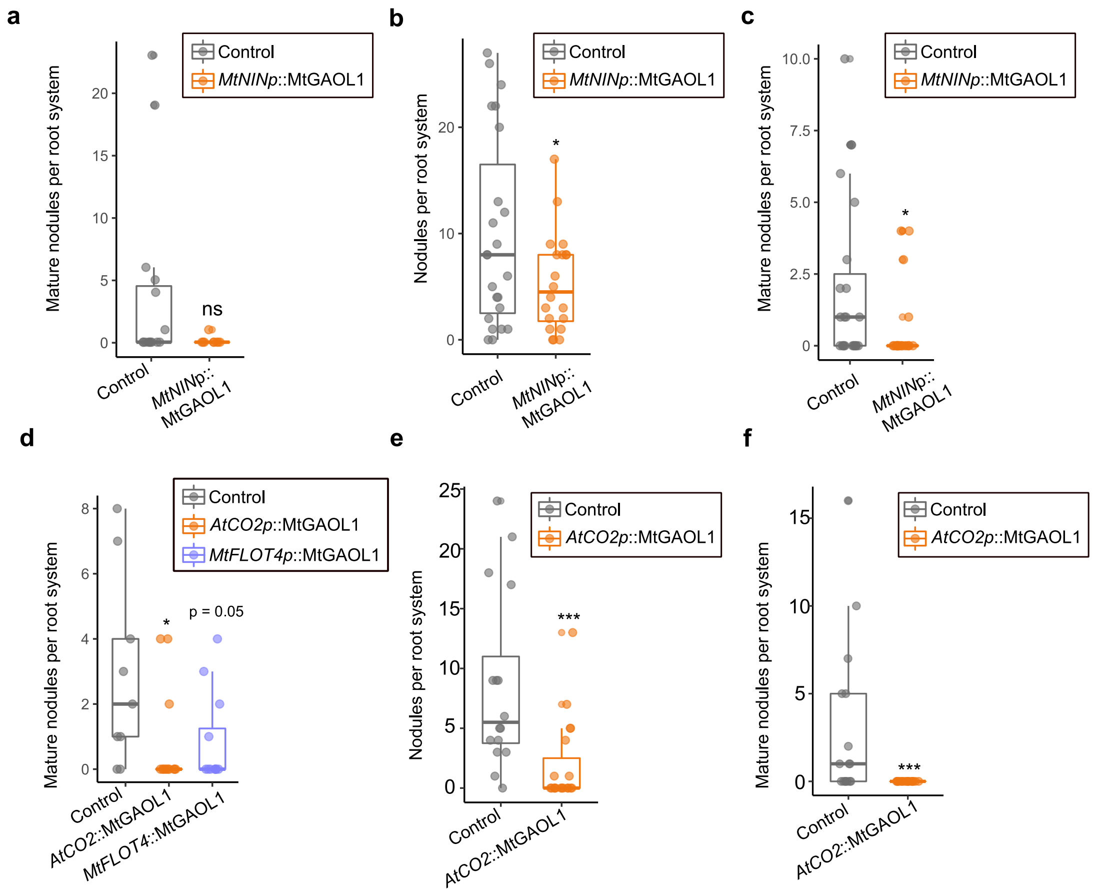

Supplementary Figure S10. Supporting information for the function of GA in developing nodule primordia cortical cells. Supports Main Figure 3.

(a) Total mature (pink) nodules per root systems from replicate displayed in Fig. 3f. Median values: control = 0, *MtNINp:*GAOL1 = 0. Boxplot center line indicates median; box limits, upper and lower quartiles; whiskers, 1.5x interquartile range; all data as points. Wilcoxon rank sum test, ns = not significant. N≥10 plants. **(b)** Total nodules per root system of second replicate of data in Fig. 3f. Boxplot center line indicates median; box limits, upper and lower quartiles; whiskers, 1.5x interquartile range; all data as points. Welch's t -test *p-value < 0.05, median values: control = 8, GAOL1 = 5. N≥ 21 plants. **(c)** Mature (pink) nodules per root system of second replicate of experiment in Fig. 3f. Boxplot center line indicates median; box limits, upper and lower quartiles; whiskers, 1.5x interquartile range; all data as points. Welch's t -test *p-value < 0.05. Median values: control = 1, *MtNINp:*GAOL1 = 0. N≥ 21 plants. **(d)** Total mature (pink) nodules per root systems from data in Fig. 3g. Boxplot center line indicates median; box limits, upper and lower quartiles; whiskers, 1.5x interquartile range; all data as points. Wilcoxon rank sum test *p-value < 0.05, p-value for *MtFLOT4p*:GAOL1 = 0.0499. Median values: control = 2, *AtCO2p:*GAOL1 = 0, *MtFLOT4p*:GAOL1 = 0. N≥10 plants. **(e)** Total nodules per root system in second replicate of data in Fig. 3g. Boxplot center line indicates median; box limits, upper and lower quartiles; whiskers, 1.5x interquartile range; all data as points. Wilcoxon rank sum test ***p-value < 0.001. Median values: control = 5.5, *AtCO2p:*GAOL1 = 0. N≥16 plants. **(f)** Mature (pink) nodules of second replicate of data in Fig. 3g. Boxplot center line indicates median; box limits, upper and lower quartiles; whiskers, 1.5x interquartile range; all data as points. Wilcoxon rank sum test ***p-value <0.001. Median values: control = 1, *AtCO2p:*GAOL1 = 0. N≥16 plants.

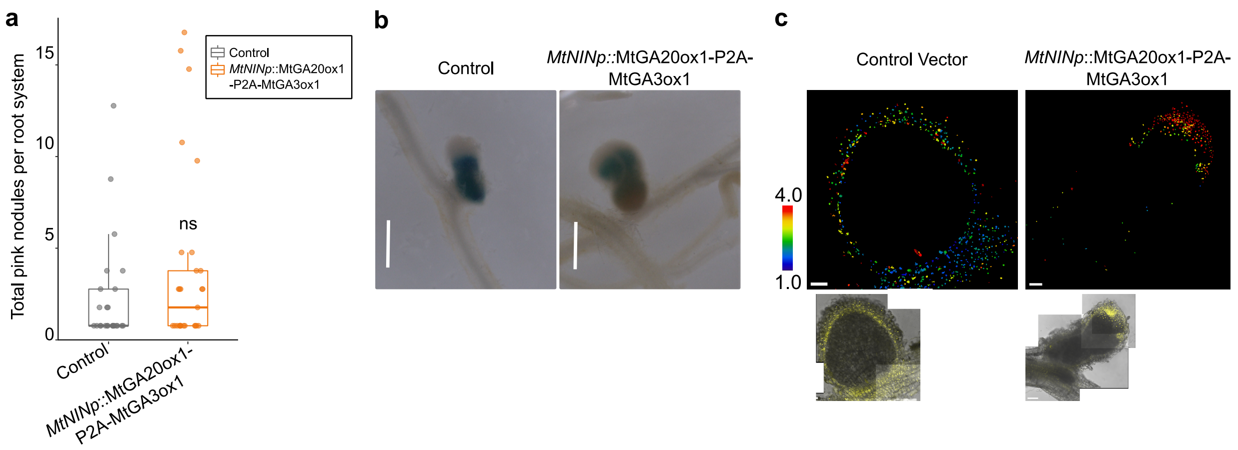

Supplementary Figure S11. Increasing GA in the nodule increases nodule size but not nodule number. Supports Main Figure 3.

(a) Total mature nodules per root system in *A. rhizogenes* roots transformed with red plasma-membrane cassette control (*AtUBQp*:PM-mScarleti) or *MtNINp:*MtGA20ox1-P2A-MtGA3ox, plasma-membrane cassette. Boxplot center line indicates median; box limits, upper and lower quartiles; whiskers, 1.5x interquartile range; all data as points. Welch's t -test, not significant. Median values: control = 0, *MtNINp*:MTGA20ox1-P2A-MtGA3ox1 = 1. N≥25 plants**. (b)** Representative images of nodules from control and *MtNINp:*MtGA20ox1-P2A-MtGA3ox1-transformed roots. Bar = 1000µm. Blue is indicative of GUS staining for Sm2011 *nifH:*GUS, which express GUS under a symbiotic-regulated operon. **(c)** Emission ratio (top panel) of *LjUBQp:*nlsGPS2 *M. truncatula* roots transformed with control or *MtNINp:*MtGA20ox1-P2A-MtGA3ox1 and YFP, brightfield (bottom panel), N=3 nodules. Bar = 100µm.

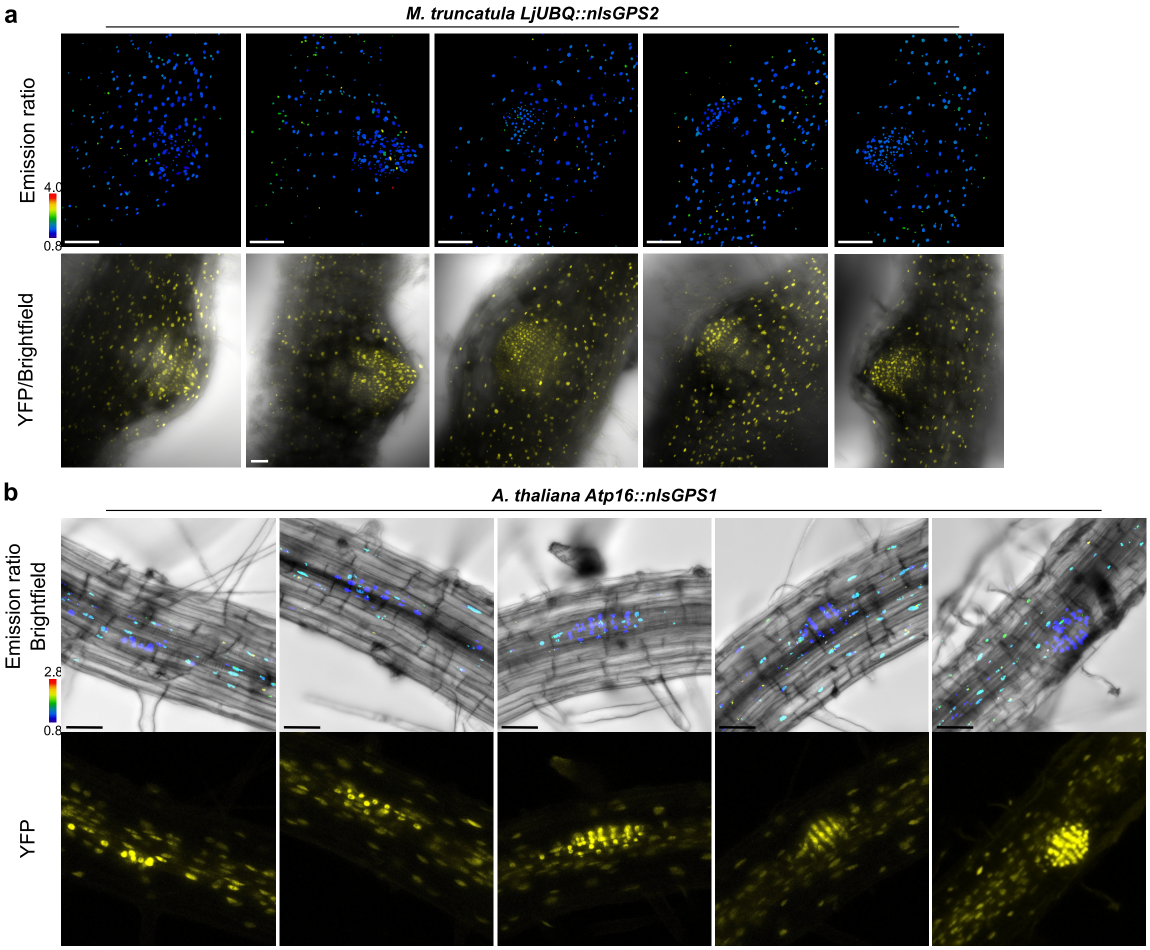

Supplementary Figure S12. GA accumulation is low in lateral roots in *M. truncatula* and *A. thaliana.* Supports Main Figure 5.

(a) Representative images of the emission ratio (top panel) and YFP control/brightfield overlay (bottom panel) of lateral root primordia from 6 day old *M. truncatula* nlsGPS2 transgenic lines grown on medium without rhizobia treatment. N=20 plants, three biological replicates. Scale bar = 100 µm. **(b)** Representative images of the emission ratio and brightfield overlay (top panel) and YFP control channel (bottom panel) of lateral root primordia from 9 day old *A. thaliana* nlsGPS1 transgenic lines. N=9 plants across 3 biological replicates. Scale Bar = 50 µm.

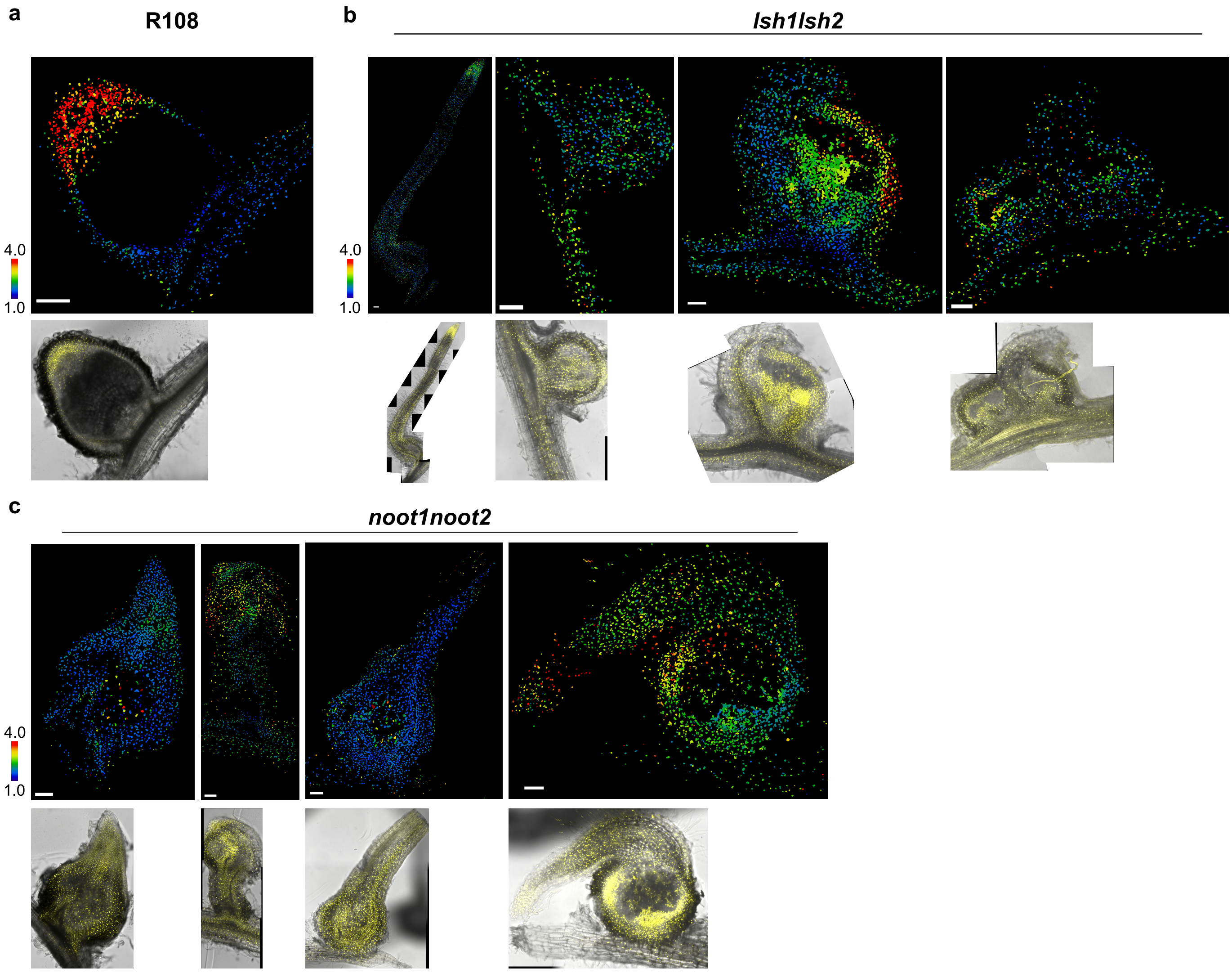

Supplementary Figure S13. Additional examples of GA accumulation in nodule development in organogenesis mutants. Supports Main Figure 5.

(a) Example emission ratio (top panel) and yellow fluorescent protein (YFP)/brightfield control channels from a 2 weeks post infection (wpi) R108 nodule. This is the same image featured in Main Fig.1 and Fig. S2. Additional 2 wpi R108 nodules can be viewed in Fig. S2. **(b)** Additional example emission ratios from *lsh1 lsh2* mutants (top panel) and their respective YFP/brightfield control channels from 2wpi nodules. **(c)** Additional example emission ratios from *noot1 noot2* mutants (top panel) and their respective YFP/brightfield control channels from 2 wpi nodules. Bar = 100µm.

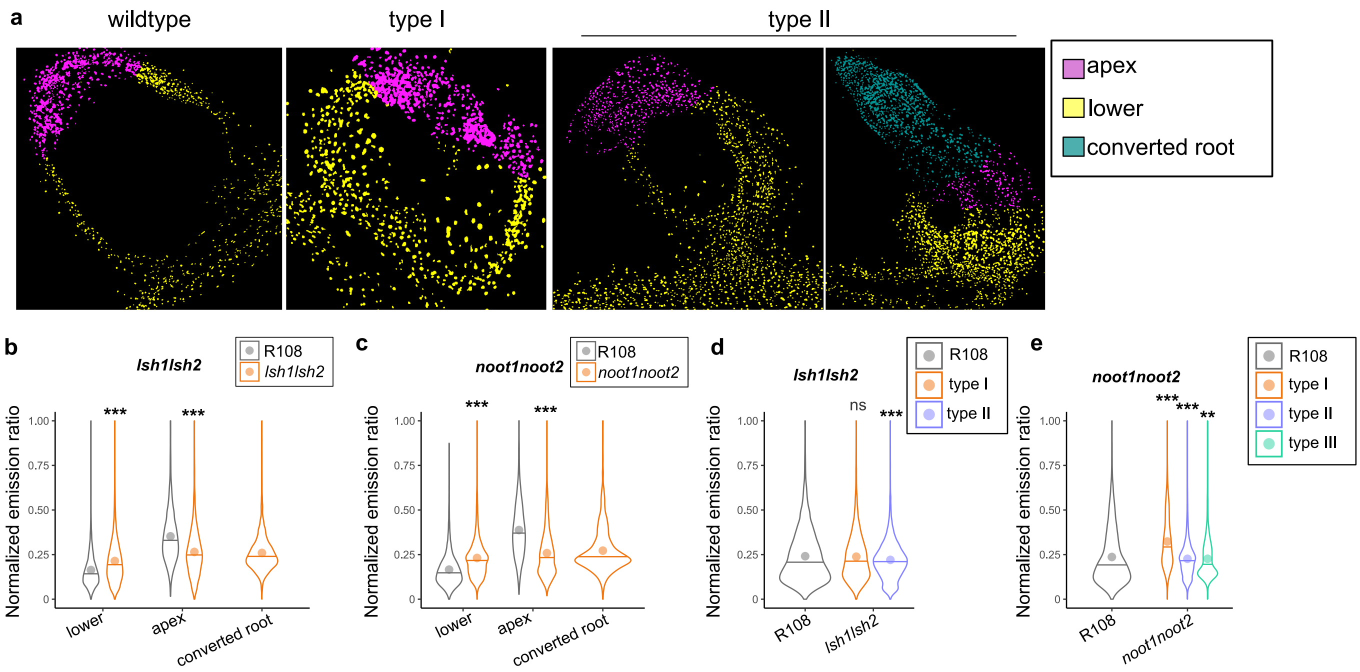

Supplementary Figure S14. Nodule organogenesis mutants have different GA accumulation from wild-type nodules. Supports Main Figure 5.

(**a**) Examples of how organ region is determined using the FRETENATOR ROI labeling map. Regions are defined using the ROI labeler on a segmented channel, blinded from the emission ratio. (**b**) Distribution of normalized emission ratio of nuclei by region between R108 and *lsh1 lsh2*; Student's T-test,***p-value <0.001. Mean values (shown as points) R108: lower = 0.1634, apex = 0.3525; median values *lsh1 lsh2*: lower = 0.2141, apex = 0.2649, converted root = 0.2594. N≥3884 from N≥12 nodules per genotype. (**c**) Distribution of normalized emission ratio of nuclei by region between R108 and *noot1 noot2*. Student's T-test, ***p-value <0.001. Median values R108: lower = 0.1659, apex = 0.3876. Mean values (shown as points) *noot1 noot2:* lower = 0.2310, apex = 0.2583, converted root = 0.2723. N≥2451 from N≥7 nodules per genotype. (**d**) Distribution of normalized emission ratio of nuclei by organ types in R108 and *lsh1 lsh2* mutants. Student's T-test, ***p-value <0.001. Mean values (shown as points): R108 = 0.2410, type I = 0.2383, type II = 0.2203. N≥9901 nuclei from N≥9 nodules per organ type. (**e**). Distribution of normalized emission ratio of nuclei by organ types in R108 and *noot1 noot2* mutants. Student's T-test, **p-value<0.01, *p-value <0.001. Mean values (shown as points): R108 = 0.2364, type I = 0.3232, type II = 0.2263, type III = 0.2265. N≥3185 nuclei from N≥3 nodules per organ type. Data shown are from three biological replicates. Emission ratio plotted in (**b-e**) are from the same dataset presented in main Figure 5.

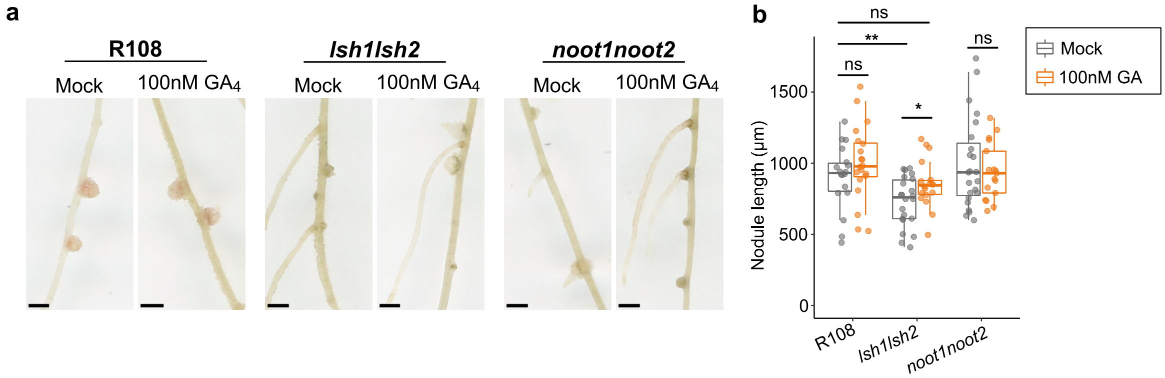

Supplementary Figure S15. GA treatment after nodule initiation of wild-type and organogenesis mutants. Supports Main Figure 6.

(a) Representative scans of R108, *lsh1 lsh2* and *noot1 noot2* at 14 dpi treated at 4 dpi with mock (70% ethanol) or 100 nM GA_4. Bar = 1.0 mm. **(b)** Quantification of nodule length at 14 dpi for plants treated with mock or 100nM GA_4. Boxplot center line indicates median; box limits, upper and lower quartiles; whiskers, 1.5x interquartile range; all data as points. Median values R108: mock = 930.45, GA_4 = 977.49; median values: *lsh1 lsh2*: mock = 759.52, GA_4 = 843.59; median values *noot1 noot2:* mock = 935.12, GA_4 = 928.57; Student's t-test *p-value <0.05, **p-value < 0.01, ns = not significant. N≥17 plants. Data shown from three biological replicates.

**Gene specific primers**					
**Insertion line**	**Gene Name**	**Primer Left 5'- 3'**	**Primer Right 5'- 3'**	**Insertion**	**Reference**
NF19087	MtGA3ox1	CTCCCCTCTCCAACAACAAAATCAG	TGATCAAGAGGGGATCCAACGAT	Tnt1-F with right Primer or TntR with left primer	Drapek et al. 2024
NF13294	MtGA3ox1	CTCCCCTCTCCAACAACAAAATCAG	TGATCAAGAGGGGATCCAACGAT	Tnt1-F with left Primer or Tnt1-R with right primer	Drapek et al. 2024
NF2717	NOOT1	GCAACAGAAACAACTAGCGA	TACACCAACCTTGAATCCAT	Tnt1-F1 with right primer	Magne et al. 2018
NF5464	NOOT2	ACTCTAAGATCTCTCTCCCTTG	GCGGAAGAGAAGATTGGAAATA	Tnt1-F1 with left primer	Magne et al. 2018
NF17203	LSH1	TGAAACCTGGAACATCTCTTG	CACCACTTTTTCTGCTACTTCA	Tnt1-R1 with left primer	Schiessl et al. 2023
NF14992	LSH2	GAATCATCGTCCACCTCTTTC	TACATTTGGAGAGGACAACACT	Tnt1-F1 with left primer	Schiessl et al. 2023
**Tnt1 Primers**					
**Primer name**		**Primer Left 5'- 3'**			**Reference**
Tnt1-F		TGTAGCACCGAGATACGGTAATTA ACAAGA			Cheng et al. 2011
Tnt1-R		CAGTGAACGAGCAGAAC CTG TG			Cheng et al. 2011
Tnt1-F1		TCCTTGTTGGATTGGTAGCCAACTTTGTTG			Cheng et al. 2011
Tnt1-R1		TGTAGCACCGAGATACGGTAATTA ACAAGA			Cheng et al. 2011

Supplementary Table S1. Primer sequences for genotyping Tnt insertion lines. Primer description, sequences, pairings (where appropriate) and references.
