# Supplementary material for: Gibberellin dynamics governing nodulation revealed using GIBBERELLIN PERCEPTION SENSOR 2 in Medicago truncatula lateral organs
Source: Plant Cell. 2024 Jul 16;36(10):4442–56. doi: 10.1093/plcell/koae201 (PMC11449112; doi:10.1093/plcell/koae201)
Supplement: koae201_Supplementary_Data [file tpc.00385.2024-s08.pdf › tpc.00385.2024-s08.pdf]

THE PLANT CELL

Root nodulation cellular gibberellin dynamics are distinct from lateral roots and governs nodule development, morphology and maturation

Colleen Drapek, Annalisa Rizza, Nadiatul Radzman-Mohd, Katharina Schiessl, Fabio Dos Santos Barbosa, Jiangqi Wen, Giles Oldroyd, and Alexander Jones

Review Timeline:

Submission Date:	06-Nov-2023
Editorial Decision:	16-Dec-2023
Resubmission Received:	24-Apr-2024
Accepted:	18-May-2024

Dr. Alexander M Jones
Sainsbury Laboratory
Cambridge, CB2 1LR
United Kingdom

Dear Dr. Jones:

Thank you for choosing to send your manuscript entitled "Root nodulation cellular gibberellin dynamics are distinct from lateral roots and govern nodule development, morphology and maturation" for consideration at The Plant Cell. Your submission has been evaluated by members of the editorial board as well as expert reviewers in your field. These experts were also given access to the manuscript of Griffiths et al., but were asked to review only this manuscript.

We regret to inform you that we are not able to recommend the publication of this manuscript in its current form. We have had input from multiple scientists and we have solicited post-review comments.

During the post-review consultation session, we also agreed that if you could address the major points raised by the reviewers by new experiments, we would welcome a resubmission.

As you can see in the reviews below, the reviewers pointed out several shortcomings, some of which can be addressed with additional experiments. These include addressing the validity of the GPS2 sensor in sectioned tissue, as well as in meristematic vs non-meristematic cells. Further, reviewers also pointed out that the argument about a feed-forward loop of cytokinin on GA accumulation during nodulation is not substantiated by the data presented, and should be removed or substantiated with further experiments.

It will be important to convince the editors and reviewers that the study adds significant new understanding(s) of mechanisms or processes, or otherwise represents a significant advance in the field, and that the major claims made are fully justified by the data presented. This includes careful consideration and explanation of the various controls used in experiments, the extent and manner of replication, and the statistical analyses used. Sampling methods and the nature of "biological replicates" should be described precisely (i.e. different plants, parts of plants, pooled tissue, independent pools of tissue, sampled at different times, etc.), along with a clear description of and rationale for any statistical analyses conducted. The reader should know exactly what was sampled; what forms the basis of the calculation of any means and other statistical variables and parameters reported. This is also necessary to ensure that proper statistical analysis was conducted.

The Plant Cell now requires authors to complete and submit an author revisions checklist upon submission of a revised manuscript. The aim of the checklist is to aid authors in preparing a high-quality manuscript, facilitate the review and assessment of revised manuscripts, and help to ensure that journal standards are maintained across the board. If your manuscript is accepted, the completed checklist will be published as supplemental material attached to the article online. Please download a copy of the checklist (a fillable PDF form) at this link, for submission with your revised manuscript: https://tpc.msubmit.net/html/Author_Revisions_Checklist.pdf.

Note also that supplemental materials should be restricted to large datasets and tables, presentation of replicates, and validation of reagents, methods, or genotypes. Any data that are used to support the main claims must be in the main manuscript. Supplemental figure legends must indicate what figure in the main manuscript is supported by the supplemental data presented.

We thank you for your interest in and support of The Plant Cell. We hope we will be able to render a more positive decision on future work.

Sincerely,

The Plant Cell Board of Editors

-- Reviewer comments:

Reviewer #1 (Comments for the Author):

Drapek et al
This interesting manuscript describes the use of a GA biosensor to analyze GA accumulation in nodules and lateral roots as

they develop. The spatial and temporal resolution provided by the biosensor is impressive and provides an entirely new view of GA during nodulation. By coupling the biosensor imaging technology with a variety of mutants and transgenics that modulate active GA levels, the authors provide several new and significant insights into the roles of GA during nodulation. The conclusion that GA is key differentiator of nodules and lateral roots is an important discovery and is mostly well supported by several lines of evidence. Also important, is the finding that nodule size is regulated by GA. I am less convinced about the strong conclusions of a feed forward loop proposed for cytokinin. Additional points are detailed below and perhaps the authors can further support this claim.

I have two technical concerns about the data that I feel the authors must address (and should be able to address fairly easily). The first is the validity of the biosensor imaging in sectioned nodule tissue. I realise that sectioning may be necessary because the nodules are thick and cannot be imaged from the outside but it is essential to provide some controls to demonstrate that GA levels are not perturbed when the nodules are cut (bioactive GA can be modulated in many ways). Perhaps imaging of an intact tissue and comparing the data with agarose-embedded sections of the same tissue would reveal whether sectioning alters GA accumulation. Hypocotyls, (as shown in the accompanying paper) might be a suitable tissue in which to do this as it appears they can be imaged intact and they could also be sectioned. The second control relates to the specificity of the sensor. In prior papers, the authors report a non-responsive GA sensor which they use to evaluate non-specific FRET differences across the cells that are being imaged. I feel that it is important to evaluate the non-responsive sensor in nodules to demonstrate that the gradients observed in nodule primordia and meristems arise because of alterations in GA and not other aspects of meristematic cells versus other cells of the nodule. One or two control experiments will provide a baseline to support all the other imaging experiments.

Overall, I am highly supportive of this manuscript. In general, the data are of high quality and novelty. I consider that it will meet the criteria required for publication in Plant Cell once the technical points mentioned above, and the additional points below, have been addressed.

Additional points to be addressed:
Figure 1. Does this show a section through the middle of the nodule? The legend indicates that the nodules were sliced into 100um sections. The position of the section is important as a section from close to the edge of the nodule will differ from a section taken through the nodule center and will show fewer meristematic cells and therefore a different interpretation. How do you confirm the position of the section and ensure that comparisons between samples are consistent? This information should be added to the materials and methods.
The methods for imaging and calculation of intensity plots are not provided. With the current information presented in the methods, it would not be possible for someone to reproduce the experiments. Details of the imaging and calculations should be presented. How are the data scaled?
Lines 321 and Figure 4C. There is no GA in the nin mutant but there are no nodules in nin so how can the cytokinin conclusion be drawn ? It seems to be a chicken and egg issue. How can you be sure that cytokinin is accumulating sufficiently in the rhizobium inoculated nin roots? Are there other data that can support the conclusion of a feed forward loop? Current support for this is not so strong.
Figure 5a shows a comparison of a 2wpi nodule and a lateral root primordium. This is interesting but this is not really a fair comparison. Lateral root and nodule primordia should be compared and then lateral root and nodule meristems should be compared. Please add comparable images.
Figure 5. The description in the text and the labeling in the figure legends is difficult to follow. The legend for Fig 5c mentions the types but not the apex, lower and converted. The labels should be described in the figure legend.
The GA accumulation in the spontaneous nodules (Figure S5) looks very different to the previous images of nodules. There are cells at the nodule edge that are higher than those in the meristematic zone. Additional data is needed to substantiate the claim that spontaneous nodules show a GA accumulation pattern similar to those of infected nodules. I am a little concerned that repetition for some of the experiments is quite low (3 nodules). This is one such experiment.
Figure 2 and S1. What are the 'mock' treatments? I was unable to find a description of this in the M and M. Please add the information to the figure legend.
Line 52 - what is meant by the phrase 'cytokinin recruitment of NIN'?
Figure S6 - The TEM images need some labeling so that readers can follow what it is that you would like to show. If the aim is to show symbiosomes, I suggest including a higher magnification image.
Is there evidence for the specificity of the CO2 promoter in M. truncatula?
Figure S10e. Is the comparison of 'lower' in R108 and noot1noot2 significantly differently (to the level of three ***)? The graphs appear identical - perhaps this a mistake with the graph?
The insertion mutants for GA3oxidase are not described. Where is this Tnt insertion and is it likely to result in a null mutation? Is there only one GA3 oxidase? I see that later in the manuscript, that there are probably two. If so, are these single mutant data useful?

Reviewer #2 (Comments for the Author):

Scope and importance of research: Drapek et al. study the dynamics of gibberellic acid (GA) during nodulation, a phytohormone previously identified as both a positive and negative regulator of this plant-rhizobia interaction. They make particular use of a novel high-sensitivity GA biosensor developed in other recent work (Griffiths et al, 2023, the accompanying manuscript) to visualise how the cellular location of GA changes over the course of nodule development. The nlsGPS2 line enabled the fine-detail of GA localisation and 'amount' to be determined in a very elegant way. The comparison of GA accumulation between lateral root development in Arabidopsis and Medicago was also very informative.

They found that GA accumulation could also be influenced by post-transcriptional regulation of GA3ox activity or depletion of GA. They concluded that GA was a positive regulator in nodule organogenesis and determinator of lateral root organogenesis and nodulation, and also found evidence for a feedforward loop involving cytokinin, then NIN regulation of local GA accumulation. The work also helps to explain previous contradictory findings related to the importance of GA as well as cytokinin in two developmental processes (lateral root development and nodulation).

I enjoyed reading this manuscript and found the experiments to be logically and conclusions well-supported using a combination of molecular and phenotypic data.

Major comments:

It made sense to study mutants for MtGA3ox1 but why not also GA20-oxidase which should be further upstream, and for which modified expression lines were studied? Adding in the rationale for the choice of mutants would be useful.

However, it was noted there were limitations in using the biosensor in the central zone of the nodule due to the oxygen requirement of bacteriods there. Do the authors think the findings obtained would be altered if there was not this issue? In the discussion (lines 450-454) it would be useful to suggest, based on understanding of the expression of other GA-related genes in central tissues, what might the effects be there.

Minor comments/typographical by lines:

Very general, but I would have liked to have more space to see the visual images compared to the graphs, e.g. in Fig.5 the phenotypic/microscopy part if very small and there is a lot of space between the graphed data - can you adjust any of these?

Lines 50-52: Introduction: you list the effects of cytokinin and say it has effects in different species but with a long list of references - please can you define the species per reference to help guide the non-specialist?

Lines 59-64: Again, splitting up the references and linking them to the genes you are introducing would help.

Lines 69-90: In the Introduction: you outline various effects of GA on different legumes but it hard to discern what the similarities/differences are: can you re-write to clarify this?

Line 140: A. thaliana spelling

Line 148: What does having a 'robust' pattern mean? Consistently observed?

Line 321: Coherent feedforward loop: it would be useful to illustrate this in a model

Line 542: Remove the 'with'

Line 634: What is a 'demonstrative image'?

Line 965: rhizobia spelling.

Fig. S6: I would re-title this as 'nodule system architecture' or similar, rather than 'further phenotypes'

Fig S10: Is there a R108 comparison set of images in the manuscript that these should be compared against, to better assess the nodulation phyenotyping and data in d-g?

We are grateful to the reviewers for their helpful critiques and suggestions as well as many positive comments. In this revision, we have addressed all comments in a response to reviewers document and made improvements to the manuscript including additional or improved figure panels and text changes highlighted in blue.

An overview of the revisions we have made:

- Added data from 5 dpi nodules of a non-responsive control
- Included additional controls for our embedding and slicing protocol (sliced 5 dpi nodules)
- Expanded on descriptions of technical details, especially on calculation of emission ratio and imaging set up
- Clarified references and sources in the text
- Streamlined the manuscript to focus on transcriptional regulators rather than cytokinin cross-talk
- Added additional points of clarification and discussion throughout as per the suggestions of the reviewers

Reviewer #1 (Comments for the Author):

Drapek et al
This interesting manuscript describes the use of a GA biosensor to analyze GA accumulation in nodules and lateral roots as they develop. The spatial and temporal resolution provided by the biosensor is impressive and provides an entirely new view of GA during nodulation. By coupling the biosensor imaging technology with a variety of mutants and transgenics that modulate active GA levels, the authors provide several new and significant insights into the roles of GA during nodulation. The conclusion that GA is key differentiator of nodules and lateral roots is an important discovery and is mostly well supported by several lines of evidence. Also important, is the finding that nodule size is regulated by GA.

We thank the reviewer for their positive comments and careful analysis of our findings.

I am less convinced about the strong conclusions of a feed forward loop proposed for cytokinin. Additional points are detailed below and perhaps the authors can further support this claim.

See response below.

I have two technical concerns about the data that I feel the authors must address (and should be able to address fairly easily). The first is the validity of the biosensor imaging in sectioned nodule tissue. I realise that sectioning may be necessary because the nodules are thick and cannot be imaged from the outside but it is essential to provide some controls to demonstrate that GA levels are not perturbed when the nodules are cut (bioactive GA can be modulated in many ways). Perhaps imaging of an intact tissue and comparing the data with agarose-embedded sections of the same tissue would reveal whether sectioning alters GA accumulation. Hypocotyls, (as shown

in the accompanying paper) might be a suitable tissue in which to do this as it appears they can be imaged intact and they could also be sectioned.

We understand this concern and appreciate the reviewers understanding of the technical limitations of deep tissue imaging. We present new data showing sliced 5 day old nodules, a pattern with which we are very familiar given our experience with whole mount imaging of non-sliced nodules. We now provide additional data showing similar GA accumulation patterns in sliced vs relevant whole mount embedded comparison samples.

We also now specify some careful precautions we took, importantly noting that the agarose embedded samples are live (not fixed) tissues in the main text and clarifying in the methods that they are imaged immediately after slicing (i.e. we do not slice in bulk and then image later).

The second control relates to the specificity of the sensor. In prior papers, the authors report a non-responsive GA sensor which they use to evaluate non-specific FRET differences across the cells that are being imaged. I feel that it is important to evaluate the non-responsive sensor in nodules to demonstrate that the gradients observed in nodule primordia and meristems arise because of alterations in GA and not other aspects of meristematic cells versus other cells of the nodule. One or two control experiments will provide a baseline to support all the other imaging experiments.

We have several experiments to address this concern, most importantly the demonstration when adding excess GA_3 to samples that the FRET biosensor is fully functional in low ratio cells. Also, adding Paclobutrazol lowers and adding the precursors GA_{12} and GA_9 raises biosensor emission ratios.

That said, we fully agree that having nlsGPS-NR lines is an excellent additional control. Unfortunately, all fluorescent lines acquired showed complete or chimeric silencing. Nonetheless, with considerable effort we acquired and analyzed nlsGPS-NR Medicago nodules and compared the distribution among the fluorescent nuclei that could be analyzed. While these lines will not be a very useful resource, we feel the additional data addresses the comment to the degree possible with available material.

Overall, I am highly supportive of this manuscript. In general, the data are of high quality and novelty. I consider that it will meet the criteria required for publication in Plant Cell once the technical points mentioned above, and the additional points below, have been addressed.

Additional points to be addressed:

Figure 1. Does this show a section through the middle of the nodule? The legend indicates that the nodules were sliced into 100um sections. The position of the section is important as a section from close to the edge of the nodule will differ from a section taken through the nodule center and will show fewer meristematic cells and therefore a different interpretation. How do you confirm the position of the section and ensure that comparisons between samples are consistent? This information should be added to the materials and methods.

We agree and have explained how we selected sections as well as further details on the embedding/slicing/selection process in the methods section.

The methods for imaging and calculation of intensity plots are not provided. With the current information presented in the methods, it would not be possible for someone to reproduce the experiments. Details of the imaging and calculations should be presented. How are the data scaled?

We take this comment seriously as we hope to provide a useful tool to the community. We have greatly expanded on this point in the Confocal image analysis and emission ratio quantification section of the methods, including settings for the FRETENATOR plugin and how we use the data generated by the plugin in R for plotting. We have also added text early in the results section describing what we are showing with segmented nuclei.

Lines 321 and Figure 4C. There is no GA in the nin mutant but there are no nodules in nin so how can the cytokinin conclusion be drawn ? It seems to be a chicken and egg issue. How can you be sure that cytokinin is accumulating sufficiently in the rhizobium inoculated nin roots? Are there other data that can support the conclusion of a feed forward loop? Current support for this is not so strong.

While this data is interesting, we see the reviewers concerns about circular logic and have decided to shelve these findings for a manuscript where the relationship between cytokinin and GA can be explored further in depth.

Figure 5a shows a comparison of a 2wpi nodule and a lateral root primordium. This is interesting but this is not really a fair comparison. Lateral root and nodule primordia should be compared and then lateral root and nodule meristems should be compared. Please add comparable images.

We agree and have added a nodule primordia comparison image.

Figure 5. The description in the text and the labeling in the figure legends is difficult to follow. The legend for Fig 5c mentions the types but not the apex, lower and converted. The labels should be described in the figure legend.

We have elaborated on these in the figure legend to make it more interpretable.

The GA accumulation in the spontaneous nodules (Figure S5) looks very different to the previous images of nodules. There are cells at the nodule edge that are higher than those in the meristematic zone. Additional data is needed to substantiate the claim that spontaneous nodules show a GA accumulation pattern similar to those of infected nodules. I am a little concerned that repetition for some of the experiments is quite low (3 nodules). This is one such experiment.

We explain in the text that the repetition is low here due to technical limits, including the recalcitrant nature of the R108 background to hairy root experiments and low penetrance of the spontaneous nodule phenotype. We now soften our language to conclude that the host can produce GA in these structures rather than say it can pattern it.

Figure 2 and S1. What are the 'mock' treatments? I was unable to find a description of this in the M and M. Please add the information to the figure legend.

Thank you for noting this oversight. We have added this to the figure legends and methods.

Line 52 - what is meant by the phrase 'cytokinin recruitment of NIN'?

We have removed the cytokinin aspect.

Figure S6 - The TEM images need some labeling so that readers can follow what it is that you would like to show. If the aim is to show symbiosomes, I suggest including a higher magnification image.

We have annotated these images to indicate symbiosomes. Unfortunately, we do not have means to increase the magnification other an artificially. Therefore, we now clarify our language to specify that it is an overview.

Is there evidence for the specificity of the CO2 promoter in M. truncatula?

The CO2 promoter is expressed in all Medicago cortex layers, as first evidenced by Rival et al 2012 https://doi.org/10.1242/dev.081620. We have made this more clear in the text by separating the cortex and epidermis references.

Figure S10e. Is the comparison of 'lower' in R108 and noot1noot2 significantly differently (to the level of three ***)? The graphs appear identical - perhaps this a mistake with the graph?

We see the viewers point here that our previous analysis of this dataset highlights a difference with very small effect size. We have now accounted for the variance of nuclei distribution among individual plants by calculating a normalized emission ratio instead (described in methods) to allow use of a parametric significance test. Plant to plant variance is already accounted for in the main figures, which plots median emission ratio for each sample.

After normalization, the distributions have changed slightly, and we find that, similar to the *lsh* mutants, the *noot* mutants have signficantly higher GA in the lower portion of the nodule with small effect size. We now mention this in the text when introducing the material with Figure 5. In the supplemental figure, we plot the mean as well as the median emission ratio and mention both values in the legend.

The insertion mutants for GA3oxidase are not described. Where is this Tnt insertion and is it likely to result in a null mutation? Is there only one GA3 oxidase? I see that later in the manuscript, that there are probably two. If so, are these single mutant data useful?

We have added in this data in a supplemental figure. Unfortunately, we could not obtain a T-DNA insertion for the MtGA3ox2 isozyme. The confounding issue of the second gene is now more clearly described in the manuscript, but individual enzymes sometimes do contribute

meaningful phenotypes and we feel the results still have merit.

Reviewer #2 (Comments for the Author):

Scope and importance of research: Drapek et al. study the dynamics of gibberellic acid (GA) during nodulation, a phytohormone previously identified as both a positive and negative regulator of this plant-rhizobia interaction. They make particular use of a novel high-sensitivity GA biosensor developed in other recent work (Griffiths et al, 2023, the accompanying manuscript) to visualise how the cellular location of GA changes over the course of nodule development. The nlsGPS2 line enabled the fine-detail of GA localisation and 'amount' to be determined in a very elegant way. The comparison of GA accumulation between lateral root development in Arabidopsis and Medicago was also very informative.

They found that GA accumulation could also be influenced by post-transcriptional regulation of GA3ox activity or depletion of GA. They concluded that GA was a positive regulator in nodule organogenesis and determinator of lateral root organogenesis and nodulation, and also found evidence for a feedforward loop involving cytokinin, then NIN regulation of local GA accumulation. The work also helps to explain previous contradictory findings related to the importance of GA as well as cytokinin in two developmental processes (lateral root development and nodulation).

I enjoyed reading this manuscript and found the experiments to be logically and conclusions well-supported using a combination of molecular and phenotypic data.

We thank the reviewer for their positive comments and careful analysis of our findings.

Major comments:

It made sense to study mutants for MtGA3ox1 but why not also GA20-oxidase which should be further upstream, and for which modified expression lines were studied? Adding in the rationale for the choice of mutants would be useful.

Unfortunately, only one of the T-DNA alleles we ordered for this gene had an insertion and, despite spraying GA at flowering onset, we could not acquire a homozygous mutant.

However, it was noted there were limitations in using the biosensor in the central zone of the nodule due to the oxygen requirement of bacteriods there. Do the authors think the findings obtained would be altered if there was not this issue? In the discussion (lines 450-454) it would be useful to suggest, based on understanding of the expression of other GA-related genes in central tissues, what might the effects be there.

We think this is an interesting point to expand on and do so in the discussion, discussing gene expression data from laser capture experiments of the infection zone.

Minor comments/typographical by lines:

Very general, but I would have liked to have more space to see the visual images compared to the graphs, e.g. in Fig.5 the phenotypic/microscopy part if very small and there is a lot of space between the graphed data - can you adjust any of these?

We agree and have enlarged the images relative to the graphs on the following figures:
Main Figures 1-6; Fig S1, S6, S11.

Lines 50-52: Introduction: you list the effects of cytokinin and say it has effects in different species but with a long list of references - please can you define the species per reference to help guide the non-specialist?

We agree this is confusing and have separated these references.

Lines 59-64: Again, splitting up the references and linking them to the genes you are introducing would help.

We agree and have separated these references out.

Lines 69-90: In the Introduction: you outline various effects of GA on different legumes but it hard to discern what the similarities/differences are: can you re-write to clarify this?

We agree and have separated out the commentary by species/GA treatment.

Line 140: A. thaliana spelling

This has been corrected – thank you.

Line 148: What does having a 'robust' pattern mean? Consistently observed?

We intend to mean consistently observed and characteristic. We have selected the word "hallmark" instead.

Line 321: Coherent feedforward loop: it would be useful to illustrate this in a model

We have now removed the cytokinin piece of the story.

Line 542: Remove the 'with'

This has been corrected – thank you.

Line 634: What is a 'demonstrative image'?

We have changed this wording to "representative."

Line 965: rhizobia spelling.

This has been corrected – thank you.

Fig. S6: I would re-title this as 'nodule system architecture' or similar, rather than 'further phenotypes'

We have re-titled as "**Nodule number and symbiosome phenotypes of *ga3ox1* mutants.**"

Fig S10: Is there a R108 comparison set of images in the manuscript that these should be compared against, to better assess the nodulation phyenotyping and data in d-g?

We now include an R108 comparison in this figure and mentioned in the legend the other examples in Fig. S2.

Dear Dr. Alexander Jones:

We are pleased to inform you that your paper entitled "Root nodulation cellular gibberellin dynamics are distinct from lateral roots and governs nodule development, morphology and maturation" has been accepted for publication in The Plant Cell, pending a final editorial review by a science editor. At this stage, your manuscript will be evaluated by a science editor with respect to its presentation of scientific content, compliance with journal policies, and presentation for a broad readership. The Plant Cell has contracted with Plant Editors (planteditors.com) to provide this service to our authors, and you will soon receive additional information on this process.

Please note that each author needs to link their ORCID identifier to their account in the system before your manuscript can be published. If any authors do not have an ORCID linked to their account, they will receive a message with a link to complete this task. Please ensure that ALL of your coauthors have completed this task as soon as possible.

ASPB offers an OPEN option that allows authors to have their online articles available for free to all users immediately upon publication. For more information about the ASPB OPEN option, refer to the Final Submission Checklist Form.

The Plant Cell and The Arabidopsis Information Resource (TAIR) are collaborating to collect functional annotation data about Arabidopsis genes from authors. This includes information about the gene's molecular function (e.g., kinase activity, ATP synthetase activity), the biological process/es it is involved in (e.g., endosperm development, threonine biosynthesis), its subcellular location (e.g., nucleus, ER), anatomical or developmental expression pattern (e.g., leaf, ovule, flower stage 10, seedling stage), or its partner in a protein-protein interaction (e.g., AT1G01010 interacts with AT1G01020).

If your paper contains results falling into one or more of these categories for Arabidopsis genes, we request that you now submit these data for inclusion in TAIR by filling in the form provided at the following URL: https://www.arabidopsis.org/doc/submit/functional_annotation/123. If you need further clarification on what types of data can be submitted please contact curator@arabidopsis.org.

Finally, we encourage your submission of artwork for the journal cover. Monthly issues will have an online cover image and selected covers will be used for posters, other promotional items, and "wallpaper" for mobile devices. For more information, refer to Cover Submission in the Instructions for Authors https://tpc.msubmit.net/cgi-bin/main.plex?form_type=display_auth_instructions.

We look forward to seeing your paper published.

Sincerely,

The Plant Cell Board of Editors

=====
IMPORTANT REMINDER: PEER REVIEW REPORTS
=====

If you opted to publish a peer review report along with your article during the original submission process, it will be prepared by the editorial staff and publicly posted with your manuscript, inside the zip file that contains any other supplemental material. As a reminder, the peer review report is a public record of all comments from editors and reviewers, as well as your prior responses, as you received them in the decision letters for each draft of your manuscript. If you agreed to publish this report and have changed your mind, or are not sure if you selected this option, please contact the editorial office as soon as possible before signing the license agreement from our publisher.

=====
REVIEWER COMMENTS
=====

Reviewer #1 (Comments for the Author):

This is reviewer 1. All of my prior positive comments still apply and so I won't repeat the intro paragraph. I appreciate the authors' careful consideration of my comments. All of my concerns have been addressed and the data now fully support the

claims made in the text. I approve publication of this revised manuscript.

I think there is one small typo in the title - govern rather than governs.

Reviewer #2 (Comments for the Author):

I was pleased to read this revision and find that it fully addresses all of my previous comments. In particular the methods are more detailed and clear, and the evaluation of the biosensor is helpful. Inconclusive aspects/findings have been pruned out and the figures are easier to read and well-explained. It was useful to be more circumspect about some findings (such as GA patterning) and the discussion also benefited from this tempering. This means that the overall manuscript is more robust.

----FOR ASPB OFFICE USE ONLY (DO NOT EDIT)----
MSID: 39195
Corresponding Author: Dr. Jones
